# Reliable estimation of membrane curvature for cryo-electron tomography

**DOI:** 10.1371/journal.pcbi.1007962

**Published:** 2020-08-10

**Authors:** Maria Salfer, Javier F. Collado, Wolfgang Baumeister, Rubén Fernández-Busnadiego, Antonio Martínez-Sánchez

**Affiliations:** 1 Department of Molecular Structural Biology, Max Planck Institute of Biochemistry, Martinsried, Germany; 2 Graduate School of Quantitative Biosciences Munich, Munich, Germany; 3 Institute of Neuropathology, University Medical Center Göttingen, Göttingen, Germany; 4 Cluster of Excellence “Multiscale Bioimaging: from Molecular Machines to Networks of Excitable Cells” (MBExC), University of Göttingen, Germany; University of Virginia, UNITED STATES

## Abstract

Curvature is a fundamental morphological descriptor of cellular membranes. Cryo-electron tomography (cryo-ET) is particularly well-suited to visualize and analyze membrane morphology in a close-to-native state and molecular resolution. However, current curvature estimation methods cannot be applied directly to membrane segmentations in cryo-ET, as these methods cannot cope with some of the artifacts introduced during image acquisition and membrane segmentation, such as quantization noise and open borders. Here, we developed and implemented a Python package for membrane curvature estimation from tomogram segmentations, which we named PyCurv. From a membrane segmentation, a signed surface (triangle mesh) is first extracted. The triangle mesh is then represented by a graph, which facilitates finding neighboring triangles and the calculation of geodesic distances necessary for local curvature estimation. PyCurv estimates curvature based on tensor voting. Beside curvatures, this algorithm also provides robust estimations of surface normals and principal directions. We tested PyCurv and three well-established methods on benchmark surfaces and biological data. This revealed the superior performance of PyCurv not only for cryo-ET, but also for data generated by other techniques such as light microscopy and magnetic resonance imaging. Altogether, PyCurv is a versatile open-source software to reliably estimate curvature of membranes and other surfaces in a wide variety of applications.

## Author summary

Membrane curvature plays a central role in many cellular processes like cell division, organelle shaping and membrane contact sites. While cryo-electron tomography (cryo-ET) allows the visualization of cellular membranes in 3D at molecular resolution and close-to-native conditions, there is a lack of computational methods to quantify membrane curvature from cryo-ET data. Therefore, we developed a computational procedure for membrane curvature estimation from tomogram segmentations and implemented it in a software package called PyCurv. PyCurv converts a membrane segmentation, i.e. a set of voxels, into a surface, i.e. a mesh of triangles. PyCurv uses the local geometrical information to reliably estimate the local surface orientation, the principal (maximum and minimum) curvatures and their directions. PyCurv outperforms well-established curvature estimation methods, and it can also be applied to data generated by other imaging techniques.

This is a *PLOS Computational Biology* Methods paper.

## Introduction

Membranes define the limits of the cells and encompass compartments within eukaryotic cells, helping to maintain specific micro-environments with different shapes and functions. Membrane curvature is important for many cellular processes, including organelle shaping, vesicle formation, scission and fusion, protein sorting and enzyme activation [1, 2]. There is a plethora of cellular mechanisms for generation, sensing and maintenance of local membrane curvature, e.g. clustering of conical lipids or transmembrane proteins, insertion of specific protein domains as well as larger scale scaffolding by e.g. cytoskeletal filaments [1, 3].

Cryo-electron tomography (cryo-ET) enables an accurate three-dimensional (3D) visualization and analysis of the subcellular architecture at molecular resolution [4–6] and is particularly well-suited to study membrane morphology. While other transmission electron microscopy (TEM) techniques may cause membrane deformations by chemical fixation and dehydration, cryo-ET allows imaging of fully hydrated vitrified cells in a close-to-native state with minimal structural perturbations [7]. The nominal resolution of tomograms can reach ∼2-4 Å per voxel, but tomograms are usually binned for membrane segmentation to enhance contrast, resulting in voxel sizes of ∼0.8-1.6 nm. Subtomogram averaging allows to routinely obtain structures in the 10-20 Å resolution range, although higher resolutions are in principle attainable [8]. Cryo-ET can be used to study membrane morphology and curvature in reconstituted preparations [9–13] and intact cells [14, 15]. We have recently employed cryo-ET to visualize peaks of extreme curvature on the cortical endoplasmic reticulum (cER) membrane facing the plasma membrane (PM). These high curvature structures are formed by Tcb proteins and help to maintain PM integrity under heat stress [16]. We have also used cryo-ET to show that polyQ-expanded huntingtin exon I fibrils induce high curvature in the endoplasmic reticulum (ER) membrane, perhaps leading to ER membrane disruption [17]. Since we lacked a method to reliably quantify membrane curvature in noisy cryo-ET data, we developed a new method, which we formally describe in this paper.

In cryo-ET, the vitreous sample is tilted around an axis inside the electron microscope, while 2D images of a cellular region of interest are acquired for each tilt. The tilt series are then computationally aligned and reconstructed into a tomogram, which is a 3D gray-value image of the cellular interior. Because in practice it is unfeasible to tilt the sample beyond ∼ ±60°, in single-tilt tomography there is a wedge of missing information in the Fourier space. This artifact, called *missing wedge* [4], causes the features to look smeared out along the electron beam direction (Z-axis), while surfaces perpendicular to the tilt axis (Y-axis) are not visible. Thus, missing membrane regions appear at the top and the bottom of both the Y- and Z-axes. Nevertheless, the missing wedge does not affect the automatically segmented membrane, the elongated regions are just omitted [18, 19]. Moreover in cryo-ET, the cells are illuminated by only a low dose of electrons, resulting in tomograms of low signal-to-noise ratio. Segmentation, i.e. voxel labeling of structural components present in tomograms, is necessary for tomogram interpretation. Available software packages can assist membrane segmentation [18, 20–22], but in most cases human supervision is still necessary due to the complexity of the cellular context and the low signal-to-noise ratio.

Currently, the interpretation of membrane segmentations is limited by the lack of computational methods to measure quantitative descriptors. Here, we quantitatively determine local curvature descriptors of cellular membranes from tomogram segmentations. A membrane can be modeled as a surface [23], so that curvature descriptors characterize its local geometry. For a surface embedded in a 3D space, principal curvatures measure the maximum and minimum bending at each point, while the principal directions define the directions of the principal curvatures as orthogonal vectors embedded on the tangent plane to the surface at each point [24]. From the principal curvatures, both extrinsic (mean) and intrinsic (Gaussian) surface curvatures can be computed for each point.

An oriented triangle mesh is the most common way to represent discrete surfaces [25]. However, triangle mesh generation from a set of voxels [26] is not trivial because of the presence of holes in membrane segmentations. Besides the errors generated during membrane segmentation, quantization noise [27] is the limiting factor for describing local membrane geometry. The term quantization noise includes here all accuracy limiting factors induced by the discretization of segmented data using binary voxels (1 membrane and 0 background). This binary discretization leads to step-wise surfaces, since surface extraction algorithms would need gray levels to achieve subvoxel precision.

Curvature estimation algorithms can be divided into three main categories: discrete, analytical and based on tensor voting. Discrete algorithms use discretized formulae of differential geometry, approximating a surface from a mesh [25, 28–31]. However, the majority of those algorithms use only a 1-ring neighborhood, i.e. triangle vertices sharing an edge with the central vertex, and therefore are not robust for coarsely triangulated, noisy surfaces [32]. An exception is [31], which uses a geodesic neighborhood of a certain size. Moreover, discrete algorithms do not directly estimate the principal directions or principal curvatures [33]. Analytical algorithms fit surfaces [32, 34] or curvature tensors [35–37] to local patches of the mesh, defined by a central vertex and a small neighborhood around it, and derive principal curvatures and directions from their model. The surface fitting algorithms are more robust to noise but more susceptible to surface discontinuities [33]. The last category of algorithms applies Medioni’s tensor voting theory [38] on a neighborhood of an arbitrary size to fit curvature tensors, increasing the robustness of principal directions and curvatures estimation for noisy surfaces with discontinuities [33, 39, 40]. However, [33] leads to wrong curvature sign estimation for non-convex surfaces, while [39, 40] were designed for point clouds instead of triangle meshes. While most of the algorithms operate on triangle vertices because the computation of distances on surfaces is straightforward, some operate on triangle faces [31, 36, 37], exhibiting a more robust behavior on irregularly tessellated and moderately noisy meshes.

Discrete curvature estimation algorithms are included in two software packages for analysis of magnetic resonance imaging (MRI) data of the human brain: the widely used FreeSurfer [41] and the newer Mindboggle [42]. Curvature of the interventricular septum in the heart from MRI was estimated in 3D using smoothing 2D spline surfaces and differential geometry operators [43]. However, those algorithms require strong smoothing of surfaces to achieve robust results, which would lead to a loss of high resolution details present in cryo-ET data.

For microscopy data, there is software to study curvature of linear cellular structures like microtubules [44], which is not applicable to surfaces. For fluorescence microscopy data, smooth point cloud surfaces of cellular membranes were reconstructed and their curvatures estimated based on local surface fitting [45]. Hoffman et al. [46] also used a local surface fitting method to estimate membrane curvature from block-face electron microscopy data. However, also these methods employ strong smoothing of surfaces, eliminating small structural details. In cryo-ET, some membrane curvature approximation methods have been already proposed [9, 14], but they only work on 2D slices and are not capable of measuring curvature on arbitrary membranes in 3D.

Here, we developed and implemented a method for robust membrane curvature estimation from tomogram segmentations. In brief, the workflow has the following steps. (1) From a segmentation, a single-layered, signed triangle mesh surface is extracted. (2) To extract the surface topology, we generate a spatially embedded graph. Graph vertices depict triangle centers and graph edges connect the centers of triangle pairs sharing an edge or a vertex. (3) Local curvature descriptors are computed for every triangle center. We propose different procedures that combine two established tensor voting-based algorithms [33, 40] but operate on triangle faces, aiming to increase the robustness to membrane geometries present in cryo-ET and to minimize the impact of quantization noise. Extensive evaluation of our algorithms and comparison with three well-established ones [30, 41, 42] on synthetic and biological surfaces proved the superiority of our approach in terms of accuracy and robustness to noise for cryo-ET and other imaging techniques.

## Materials and methods

### Cryo-ET data collection and segmentation

As real-world test input files for PyCurv, in this study we used membrane segmentations from *in situ* cryo-ET data collected from vitrified cells: a human HeLa cell [17], yeast *Saccharomyces cerevisiae* (EMD-10767 and EMD-10765) and a primary mouse neuron (EMD-10766). The cells were milled down to 150-250 nm thick lamellas using cryo-focused ion beam [16, 54] and imaged using a Titan Krios cryo-electron microscope (FEI), equipped with a K2 Summit direct electron detector (Gatan), operated in dose fractionation mode. Tilt series were recorded using SerialEM software [55] at magnifications of 33,000 X (pixel size of 4.21 Å) for the HeLa cell and the mouse neuron and 42,000 X (pixel size of 3.42 Å) for yeast, typically from -50° to +60° with increments of 2°. The K2 frames were aligned using K2Align software [56]. Tilt series were aligned using patch-tracking and weighted back projection provided by the IMOD software package [57]. The tomograms were binned 4 times to improve contrast prior to segmentation, thus the voxel size of the final segmentations was 1.684 nm (HeLa cell and mouse neuron) and 1.368 nm (yeast). The contrast of one tomogram of yeast (EMD-10767) was enhanced prior to segmentation using an anisotropic filter [58], while the contrast of the other tomogram of yeast (EMD-10765) and the one of the mouse neuron was enhanced using a deconvolution filter executed in MATLAB (MathWorks) using the functionalities of the TOM toolbox [59]. Membrane segmentations were generated automatically from tomograms using TomoSegMemTV [18] using parameters s = 10 and t = 0.3 (HeLa cell), s = 12 and t = 4 (yeast) and s = 10 and t = 3 (mouse neuron) and further refined manually using Amira Software (ThermoFisher Scientific). The lumen of membrane compartments was then filled manually.

### Data preprocessing algorithms

**Fig 1:**
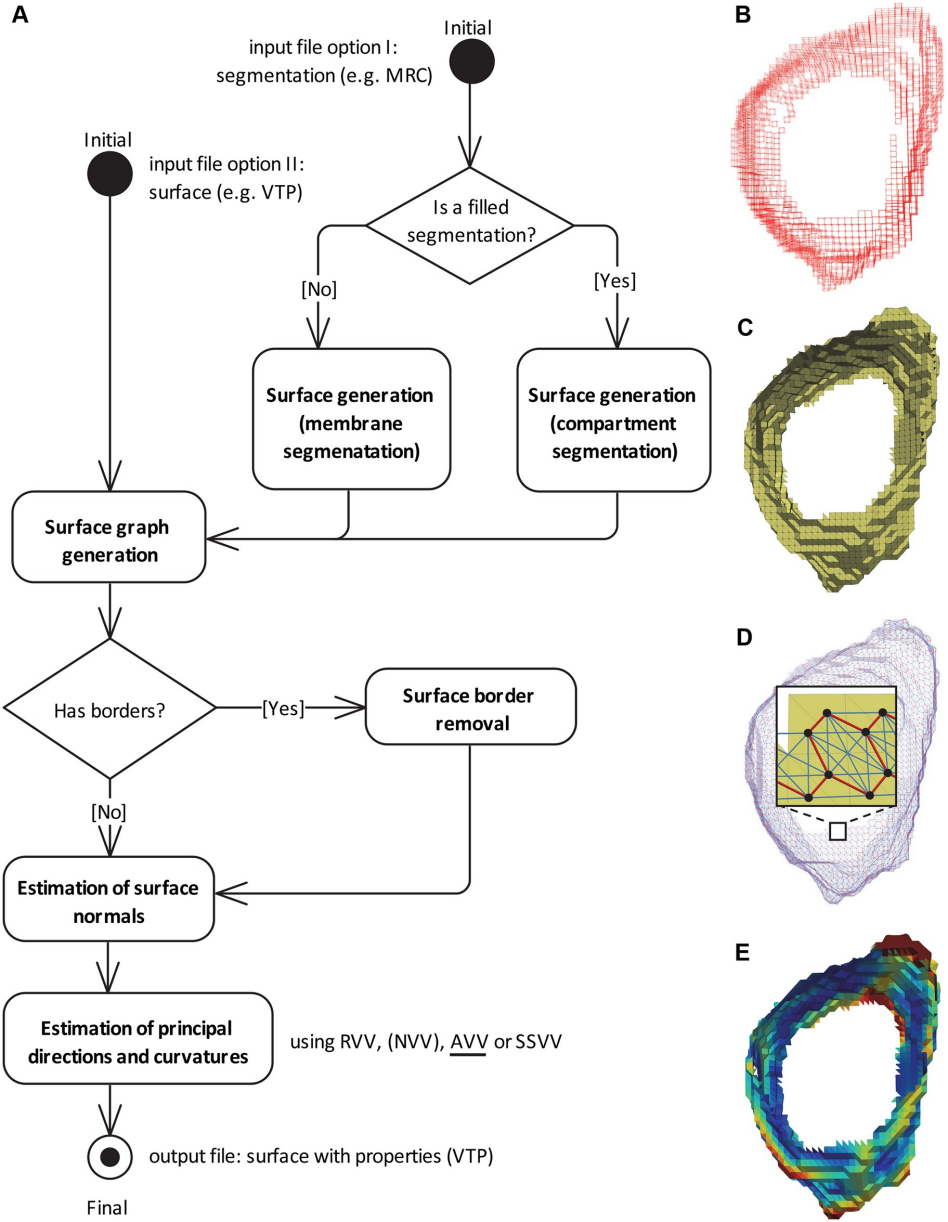
PyCurv workflow. (**A**) UML activity diagram of the PyCurv workflow. If the input segmentation (in e.g. MRC format) is filled, the surface is generated using the *compartment segmentation*, otherwise using the *membrane segmentation* algorithm. This step is omitted if the input is a surface (in e.g. VTP format). From the surface, a graph is generated. If the graph has surface borders, they are removed. Then, surface normals are estimated at each triangle center. Finally, principle directions and curvatures are estimated and different combined curvature measures calculated using one of the tensor voting-based algorithms: RVV (Regular Vector Voting), NVV (Normal Vector Voting, only for evaluation), AVV (Augmented Vector Voting, default algorithm) or SSVV (Surface Sampling Vector Voting). The output is a surface with all the calculated values stored as triangle properties (VTP format). All the processing steps (rounded rectangles) are implemented in PyCurv. (**B**) Voxels of a segmentation of a vesicle from a cryo-electron tomogram of a human HeLa cell [17]. (**C**) A surface (triangle mesh) generated from the membrane segmentation shown in (A). (**D**) Surface graph generated from the surface shown in (B); the inset shows a magnified region of the graph mapped on top of the triangle mesh (triangles: yellow, graph vertices: black dots, strong edges: red lines, weak edges: light blue lines). (**E**) The output surface with estimated normals, principal directions and curvatures as well as several combined curvature measures. Here, curvedness is shown. See also the video in S1 Video.

The first steps of the PyCurv workflow (Fig 1A) are the conversion of the input segmentation into a surface and the extraction of its associated graph.

#### Surface generation

A surface can be extracted using PyCurv from two types of input segmentations, a *membrane segmentation* or a *compartment segmentation*. This step is not required if the input is directly a surface (Fig 1A).

**Fig 2:**
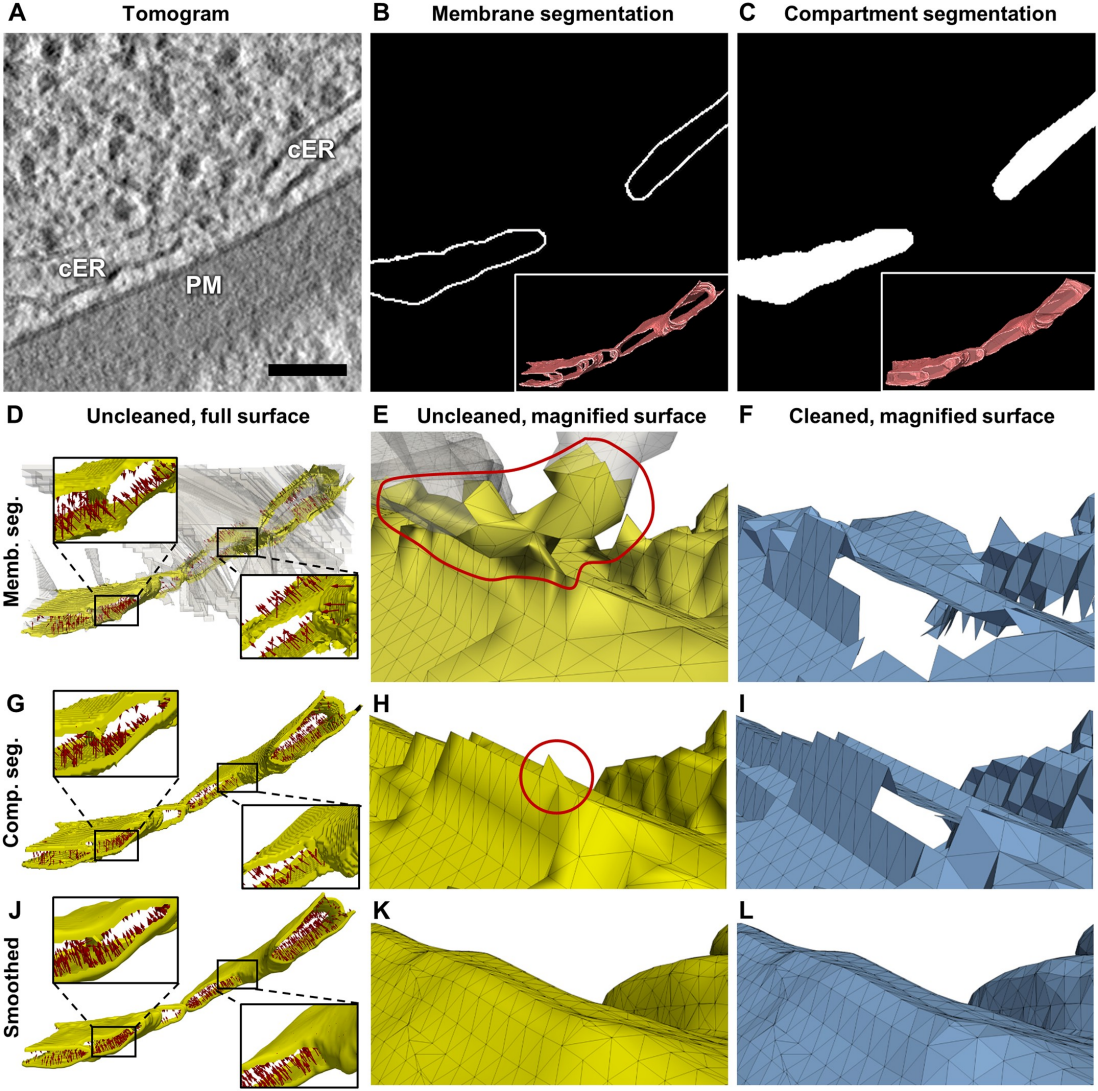
Surface generation from *membrane* and *compartment segmentations*. (**A**) A filtered tomographic slice showing the cortical endoplasmic reticulum (cER) and plasma membrane (PM) of a yeast cell (scale bar: 100 nm). Panels (B-C) show the same slice as in (A) with (**B**) the *membrane segmentation* of the cER and (**C**) the *compartment segmentation* of the cER; the insets show 3D renderings of the full segmentations (including all tomographic slices). Panels (D-F) show a surface generated from the cER *membrane segmentation* shown in (B): (**D**) The unmasked artefactual surface is shown in transparent white. The masked but uncleaned surface is shown in yellow with normals (every 100th) as red arrows. Some of the normals erroneously point outside the cER lumen (see right inset). (**E**) A different view of the uncleaned surface shown in (D), magnified. The red line marks an artifact. (**F**) The same magnified view as in (E) showing the cleaned surface in blue with a hole resulting from removing the artifact shown in (E). Panels (G-L) show surfaces generated using the *compartment segmentation* shown in (C), (G-I) without and (J-L) with Gaussian smoothing; the views are the same as in panels (D-F) column-wise: (**G**, **J**) Using the *compartment segmentation*, all normals point inside the cER lumen (see the insets). (**H**) Without smoothing, triangles sticking out (red circle) in the uncleaned surface lead to a hole in the cleaned surface shown in (**I**). (**K**-**L**) The cleaned smoothed surface is free from artifacts. The tomogram and segmentation are deposited in EM Data Bank (EMD-10767). See also the video in S1 Video.

Using the *membrane segmentation* surface generation algorithm, the segmented membrane of interest (Fig 2B) from the binned tomogram (Fig 2A) was used as the input for an algorithm [26] that reconstructs signed, single-layered triangle-mesh surfaces from an unorganized set of points, here the membrane voxels (Fig 1B). This algorithm was designed for closed surfaces without boundaries. However, most segmented membranes in cryo-ET are open, e.g. due to noise or missing wedge artifacts. Attempting to close the surface, the algorithm generated large artefactual surface regions beyond the segmentation (Fig 2D, transparent white). These regions were largely discarded by applying a mask with the membrane segmentation (Fig 2D, yellow). Since the masking was done with a distance threshold of three voxels in order to bridge upon small holes in the segmentation, additional three voxels-wide border remained. This additional border was removed in the final cleaning step (see Surface graph generation). We use the convention that normal vectors ("normals") point inwards in a convex surface. However, since membrane segmentations have boundaries, the algorithm [26] sometimes mistakenly initiates normals on both sides (Fig 2D, red arrows). As a result, ridge-like patches appear along the surface (Fig 2E), leading to holes in the cleaned surface (Fig 2F). In some cases, the surface reconstruction can be improved by closing small holes in the segmentation using morphological operators.

The *compartment segmentation* surface generation algorithm requires additional segmentation of the inner volume of a compartment enclosed by a membrane (Fig 2C). This unequivocally defines the orientation of the membrane by closing its holes. After joining the membrane and its inner volume masks, we generate an isosurface around the resulting volume using the Marching Cubes algorithm [47]. Finally, we apply a mask using the original membrane segmentation to keep only the surface region going through the membrane (again, except for the additional border that is cleaned in the end). The surface orientation is recovered perfectly in our experiments (Fig 2G). In some cases, especially where the membrane segmentation was manually refined, Marching Cubes produces triangles standing out perpendicularly to the surface (Fig 2H), leading to holes in the cleaned surface (Fig 2I). To correct those artifacts and exploit the subvoxel precision offered by Marching Cubes, the compartment segmentation mask was slightly smoothed using a Gaussian kernel with *σ* = 1 voxel before extracting the surface (Fig 2J–2L).

In summary, although compartment segmentations require more human intervention, they ensure smoother and well oriented surfaces. Thus, we choose this algorithm as the default for the subsequent data processing.

#### Surface graph generation

Curvature is a local property. Thus, for a triangle-mesh surface, curvature has to be estimated using a local neighborhood of triangles. If the neighborhood is too small, one would measure only noise created by the steps between voxels. If the neighborhood is too large, one would underestimate the curvature.

To estimate geodesic distances within membrane surfaces, we use the graph-tool python library [48] to map the triangle mesh (Fig 1A and 1C) into a spatially embedded graph, here referred as *surface graph*. First, graph vertices are associated to triangle centroid coordinates. Second, pairs of triangles sharing two triangle vertices are connected by *strong* edges, while those sharing only one triangle vertex are connected by *weak* edges (Fig 1D). To approximate the shortest paths along the surface between the centers of a source triangle and a target triangle, the graph is traversed starting from the source vertex along all its edges until the target vertex is found, using the Dijkstra algorithm [49]. Using both strong and weak edges increases the number of possible paths and thus improves the estimation of the shortest path. The geodesic distance is computed by summing up the lengths of the edges comprising the shortest path.

Another application of the surface graph is to remove surface borders to avoid wrong curvature estimations in these regions. Using the surface graph, we can detect triangles at borders because they have less than three strong edges. Then, triangles up to a certain geodesic distance from the border can be found and filtered out from the surface.

### Curvature estimation algorithms

We estimate membrane curvature from surface graphs (Fig 1A). This algorithm combines two previously published algorithms that are based on tensor voting and curvature tensor theory [33, 40], to increase the precision of curvature estimation for noisy surfaces. To estimate principal curvatures, principal directions have to be estimated. For the estimation of principal directions, surface normals are required. Surface normals are robustly estimated by averaging normals of triangles within a geodesic neighborhood.

#### Parameters defining the geodesic neighborhood

**Fig 3:**
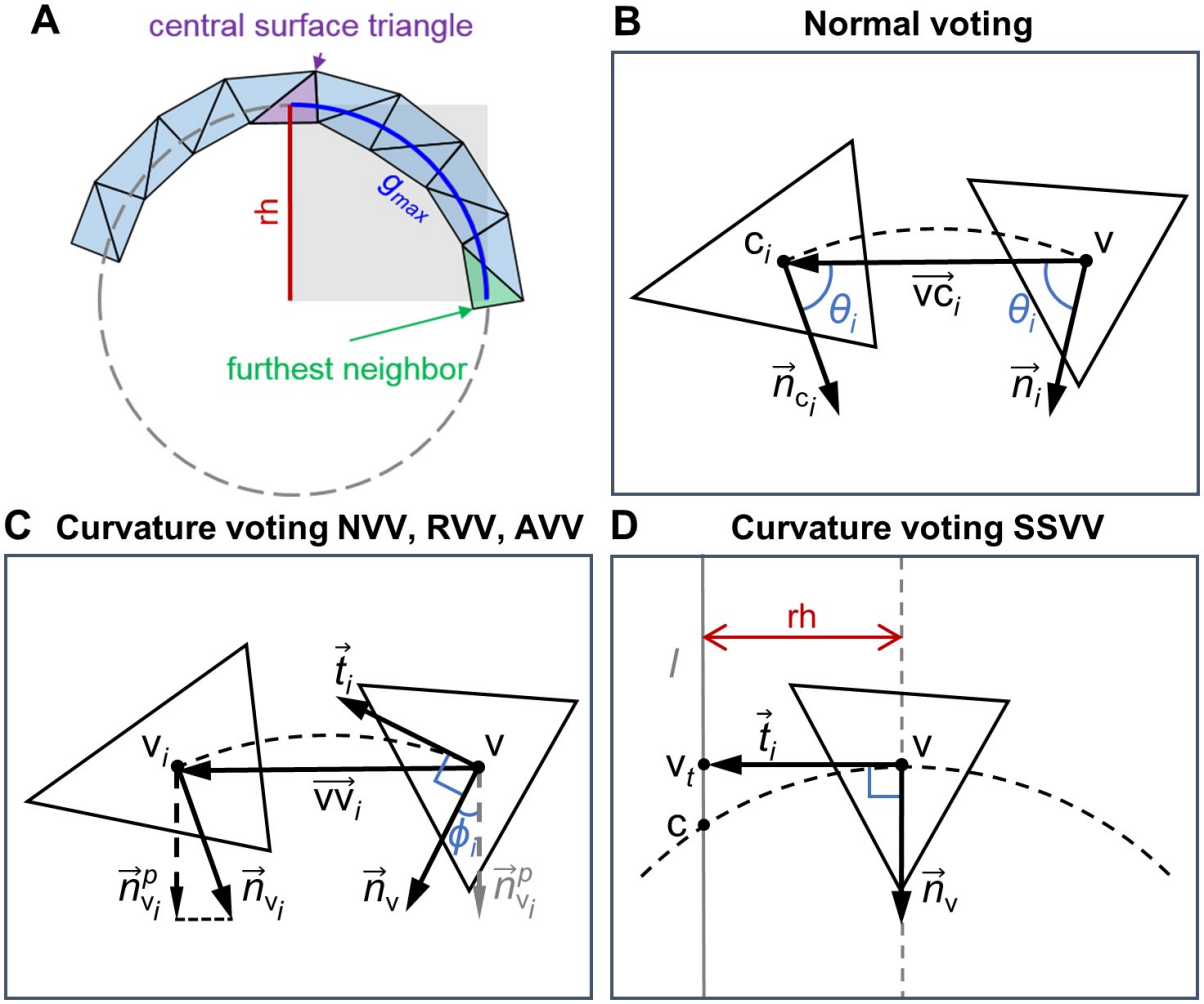
Neighborhood parameters and voting geometry. (**A**) Schematic illustrating the rh and *g*_*max*_ parameters. *g*_*max*_ is one quarter of the circle perimeter with radius equal to rh. *g*_*max*_ defines the maximum geodesic distance from a surface triangle center to the centers of its neighboring triangles, approximated by the shortest path along the edges of the surface graph. (**B**) Collection of normal votes in all proposed algorithms based on [33]. The rectangle denotes the plane containing the circular arc (dashed line) between the neighboring triangle centers v and c_*i*_, the normal vector ${\vec{n}}_{{\text{c}}_{i}}$ at c_*i*_ and the normal vote ${\vec{n}}_{i}$ at v. (**C**) Collection of curvature votes for the NVV, RVV and AVV algorithms based on [33]. The rectangle denotes the arc plane containing the triangle center v, its estimated normal ${\vec{n}}_{\text{v}}$, its tangent ${\vec{t}}_{i}$ towards neighboring triangle center v_*i*_ and the projection ${\vec{n}}_{{\text{v}}_{i}}^{p}$ of the estimated normal ${\vec{n}}_{{\text{v}}_{i}}$ at v_*i*_. (**D**) Collection of curvature votes in the SSVV algorithm based on [40]. The rectangle denotes the plane containing the tangent vector ${\vec{t}}_{i}$ at v of length = rh, ending with the point v_*t*_, and the normal vector ${\vec{n}}_{\text{v}}$ at v. The line *l*, crossing v_*t*_ and parallel to ${\vec{n}}_{\text{v}}$, intersects the surface (dashed) at point c.

Similarly to [40], here we define a radius_hit (rh) parameter to approximate the highest curvature value we can estimate reliably, i.e. rh^-1^. For each surface triangle center, we define its local neighborhood as

$$\begin{array}{c} g_{max}=\frac{\pi \cdot \text{rh}}{2}, \end{array}$$ (1)

where *g*_*max*_ defines the maximum geodesic distance. In Eq 1, *g*_*max*_ is approximated by one quarter of a circle perimeter with radius equal to rh (Fig 3A).

#### Estimation of surface normals

Normals computed directly from the triangle mesh are corrupted by quantization noise. To avoid this, we have adapted the first step of the algorithm proposed in [33], but estimating the normals for each triangle center instead of defining new normals at each triangle vertex.

For each triangle centroid (or graph vertex) v, the normal votes of all triangles within its geodesic neighborhood are collected and the weighted covariance matrix sum *V*_v_ is calculated. More precisely, a normal vote ${\vec{n}}_{i}$ of a neighboring triangle (whose center c_*i*_ is lying within *g*_*max*_ of vertex v) is calculated using the normal ${\vec{n}}_{{\text{c}}_{i}}$ assigned to this triangle:

$$\begin{array}{c} {\vec{n}}_{i}={\vec{n}}_{{\text{c}}_{i}}+2\;\text{cos}\;\theta_{i}\frac{{\vec{\text{vc}}}_{i}}{\|{\vec{\text{vc}}}_{i}\|}, \end{array}$$ (2)

where $\text{cos}\;\theta_{i}=-\frac{{\vec{n}}_{{\text{c}}_{i}}^{t}{\vec{\text{vc}}}_{i}}{\|{\vec{\text{vc}}}_{i}\|}$, ${\vec{n}}_{{\text{c}}_{i}}^{t}$ is the transposed vector ${\vec{n}}_{{\text{c}}_{i}}$, ${\vec{\text{vc}}}_{i}={\text{c}}_{i}-\text{v}$ and 0 ≤ *θ*_*i*_ ≤ *π*. This formula fits a smooth curve from c_*i*_ to v, allowing the normal vote ${\vec{n}}_{i}$ to follow this curve, so that the angle *θ*_*i*_ between ${\vec{n}}_{i}$ and ${\vec{\text{vc}}}_{i}$ is equal to the angle between ${\vec{n}}_{{\text{c}}_{i}}$ and -${\vec{\text{vc}}}_{i}$. According to the perceptual continuity constrain [38], the most appropriate curve is the shortest circular arc (Fig 3B). Then, each vote is represented by a covariance matrix $V_{i}={\vec{n}}_{i}^{t}{\vec{n}}_{i}$, and votes from the geodesic neighborhood are collected as a weighted matrix sum *V*_v_:

$$\begin{array}{c} V_{\text{v}}=\sum w_{i}V_{i}, \end{array}$$ (3)

where *w*_*i*_ is a weighting term calculated as follows:

$$\begin{array}{c} w_{i}=\frac{a_{i}}{a_{max}}exp\left(-\frac{g_{i}}{\sigma }\right). \end{array}$$ (4)

The weight of the vote of a neighboring triangle increases linearly with its surface area *a*_*i*_, but decreases exponentially with its geodesic distance *g*_*i*_ to v. *a*_*max*_ is the area of the largest triangle in the whole surface and *σ* is an exponential decay parameter, which is set to fulfill 3*σ* = *g*_*max*_, so that votes beyond the geodesic neighborhood have almost no influence and can be ignored.

The votes collected into the matrix *V*_v_ are used for estimating the correct normal vector for the triangle represented by vertex v. This is done by eigen-decomposition of *V*_v_, which generates three real eigenvalues *e*_1_ ≥ *e*_2_ ≥ *e*_3_ with corresponding eigenvectors ${\vec{e}}_{1}$, ${\vec{e}}_{2}$ and ${\vec{e}}_{3}$. The normal direction is equal in its absolute value to that of the first eigenvector. During construction of *V*_v_, the sign of normal votes is lost when *V*_*i*_ is computed. The correct orientation can be recovered from the original normal $\vec{n}$, as the original surface was already oriented. Therefore, the estimated normal is correctly oriented by:

$$\begin{array}{c} {\vec{n}}_{\text{v}}=\{\begin{array}{cc} {\vec{e}}_{1} & \text{if}\;cos({\vec{n}}^{t}{\vec{e}}_{1})>cos(-{\vec{n}}^{t}{\vec{e}}_{1})\\ -{\vec{e}}_{1} & \text{otherwise}. \end{array} \end{array}$$ (5)

#### Estimation of principal directions and curvatures

For each graph vertex v, we use the estimated normals ${\vec{n}}_{{\text{v}}_{i}}$ of its geodesic neighbors v_*i*_ in order to cast curvature votes. The curvature votes are summed into a curvature tensor. The resulting curvature tensor is decomposed to find the principal directions and curvatures at vertex v. Below, we describe the basic curvature estimation algorithm as an adaptation of [33] and [40].

Each neighboring vertex v_*i*_ casts a vote to the central vertex v, where the votes are collected into a 3x3 symmetric matrix *B*_v_ [35]:

$$\begin{array}{c} B_{\text{v}}=\frac{1}{2\pi }\sum w_{i}\kappa_{i}{\vec{t}}_{i}{\vec{t}}_{i}^{\;t}. \end{array}$$ (6)

For each v_*i*_, three variables are computed:

1. Weight *w*_*i*_ depending on the geodesic distance between v_*i*_ and v, as defined in Eq 4 but without normalizing by relative triangle area:
  $$\begin{array}{c} w_{i}=exp\left(-\frac{g_{i}}{\sigma }\right). \end{array}$$ (7)
  Also, all weights around the vertex v are constrained by ∑*w*_*i*_ = 2*π*.
2. Tangent ${\vec{t}}_{i}$ from v in the direction of the arc connecting v and v_*i*_ (using the estimated normal ${\vec{n}}_{\text{v}}$ at v) (Fig 3C):
  $$\begin{array}{c} {\vec{t}}_{i}=\frac{{\vec{t^{\prime }}}_{i}}{\|{\vec{t^{\prime }}}_{i}\|},\;{\vec{t^{\prime }}}_{i}={\vec{\text{vv}}}_{i}-\left({\vec{n}}_{\text{v}}^{t}{\vec{\text{vv}}}_{i}\right){\vec{n}}_{\text{v}}. \end{array}$$ (8)
3. Normal curvature *κ*_*i*_ [40]:
  $$\begin{array}{c} |\kappa_{i}|=\frac{|2\;\text{cos}\;\frac{\pi -\phi_{i}}{2}|}{\|{\vec{\text{vv}}}_{i}\|}, \end{array}$$ (9)
  where *ϕ*_*i*_ is the turning angle between ${\vec{n}}_{\text{v}}$ and the projection ${\vec{n}}_{{\text{v}}_{i}}^{p}$ of ${\vec{n}}_{{\text{v}}_{i}}$ onto the arc plane (formed by v, ${\vec{n}}_{\text{v}}$ and v_*i*_). The following calculations lead to *ϕ*_*i*_:
  $$\begin{array}{c} \begin{array}{c} {\vec{p}}_{i}={\vec{n}}_{\text{v}}\times {\vec{t}}_{i},\;{\vec{n}}_{{\text{v}}_{i}}^{p}={\vec{n}}_{{\text{v}}_{i}}-\left({\vec{p}}_{i}^{\;t}{\vec{n}}_{{\text{v}}_{i}}\right){\vec{p}}_{i}\\ \text{cos}\;\phi_{i}=\frac{{\vec{n}}_{\text{v}}^{t}{\vec{n}}_{{\text{v}}_{i}}^{p}}{\|{\vec{n}}_{i}\|}. \end{array} \end{array}$$ (10)

For surface generation, we use the convention that normals point inwards in a convex surface. Then, the curvature is positive if the surface patch is curved towards the normal and negative otherwise. Therefore, the sign of *κ*_*i*_ is set by:

$$\begin{array}{c} \kappa_{i}=-{\vec{t}}_{i}^{\;t}{\vec{n}}_{{\text{v}}_{i}}^{p}\left|\kappa_{i}\right|. \end{array}$$ (11)

For a vertex v and its calculated matrix *B*_v_, we calculate the principal directions, maximum ${\vec{t}}_{1}$ and minimum ${\vec{t}}_{2}$, and the respective curvatures, *κ*_1_ and *κ*_2_, at this vertex. This is done using eigen-decomposition of *B*_v_, resulting in three eigenvalues *b*_1_ ≥ *b*_2_ ≥ *b*_3_ and their corresponding eigenvectors ${\vec{b}}_{1},$ ${\vec{b}}_{2}$ and ${\vec{b}}_{3}$. The eigenvectors ${\vec{b}}_{1}$ and ${\vec{b}}_{2}$ are the principal directions. The principal curvatures are found with linear transformations of the first two eigenvalues [35]:

$$\begin{array}{c} \begin{array}{c} \kappa_{1}=3b_{1}-b_{2}\\ \kappa_{2}=3b_{2}-b_{1}. \end{array} \end{array}$$ (12)

The smallest eigenvalue *b*_3_ has to be close to zero and the corresponding eigenvector ${\vec{b}}_{3}$ has to be similar to the normal ${\vec{n}}_{\text{v}}$ [33].

#### Algorithm variants

We implemented the following algorithm variants within PyCurv.

*Vector Voting (VV)*: Estimation of surface normals algorithm, which is the same for all our algorithms listed below.

*Regular Vector Voting (RVV)*: Estimation of principal directions and curvatures algorithm described above. Modifications of this algorithm were implemented to determine the best solution for cryo-ET:

*Normal Vector Voting (NVV)*: In [33], curvature is computed as the turning angle *ϕ*_*i*_ divided by arc length between the vertices v and v_*i*_, which is the geodesic distance between them, *g*_*i*_:

$$\begin{array}{c} \kappa_{i}=\frac{\phi_{i}}{g_{i}}. \end{array}$$ (13)

However, this definition of *κ*_*i*_ with the sign according to our normals convention (Eq 11) lead to erroneous eigenvalue analysis of *B*_v_. The eigenvalue analysis was only successful for *κ*_i_ > 0, leading to wrong curvature sign estimation for non-convex surfaces (see Section Estimation of the curvature sign).

*Augmented Vector Voting (AVV)*: Here, the weights of curvature votes, prioritizing neighbors with a closer geodesic distance to the central triangle vertex v, are normalized by relative triangle area as for normal votes using Eq 4 instead of Eq 7.

*Surface Sampling Vector Voting (SSVV)*: We implemented the algorithm GenCurvVote from [40] to estimate the principal directions and curvatures. While RVV, NVV and AVV use all points within the geodesic neighborhood of a given surface point v, in SSVV only eight points on the surface are sampled using rh. For this, an arbitrary tangent vector ${\vec{t}}_{i}$ at v with length equal to rh is first generated, creating a point v_*t*_ in the plane formed by this tangent and the normal ${\vec{n}}_{\text{v}}$ at v (Fig 3D). Then, a line *l* crossing v_*t*_ and parallel to the normal ${\vec{n}}_{\text{v}}$ is drawn and its intersection point c with the surface is found. The tangent is rotated seven times around the normal by $\frac{\pi }{4}$ radians, generating another seven intersection points. Each vote is weighted equally, thus Eq 6 simplifies to:

$$\begin{array}{c} B_{\text{v}}=\frac{1}{8}\sum \kappa_{i}{\vec{t}}_{i}{\vec{t}}_{i}^{\;t}. \end{array}$$ (14)

The output of these curvature estimation algorithms comprises the surface with corrected normals, estimated principal directions and curvatures as well as different combined curvature measures: mean curvature *H* (Eq 15), Gaussian curvature *K* (Eq 16), curvedness *C* (Eq 17) and shape index *SI* (Eq 18) [50]. All these measures are stored as triangle properties in the VTP surface output file that can be viewed using e.g. the free visualization tool ParaView [51] (Fig 1E).

$$\begin{array}{c} H=\frac{\kappa_{1}+\kappa_{2}}{2} \end{array}$$ (15)

$$\begin{array}{c} K=\kappa_{1}\kappa_{2} \end{array}$$ (16)

$$\begin{array}{c} C=\sqrt{\frac{\kappa_{1}^{2}+\kappa_{2}^{2}}{2}} \end{array}$$ (17)

$$\begin{array}{c} SI=\frac{2}{\pi }atan\frac{\kappa_{1}+\kappa_{2}}{\kappa_{1}-\kappa_{2}} \end{array}$$ (18)

The complete workflow of our method including the input, processing steps and output is shown as an UML (Unified Modeling Language) activity diagram in Fig 1A. See also the video in S1 Video.

#### Other algorithms

We used the following alternative curvature estimation algorithms available in other software packages for comparison to our algorithms.

*VTK* [30]: The Visualization Toolkit (VTK) calculates curvature per triangle vertex using only its adjacent triangles and applying discrete differential operators [25]. In order to be able to compare VTK to our tensor voting-based algorithms operating on triangles, we average the values of each curvature type at three triangle vertices to obtain one value per triangle. VTK does not estimate principal directions.

*FreeSurfer* [52]: FreeSurfer’s function mris_curvature_stats [41] estimates mean, Gaussian and principal curvatures, curvedness as well as other local and global derived curvature measures per triangle vertex, based on osculating circle fitting. FreeSurfer fails on surface edges and holes, so it cannot be applied to a cylindrical surface.

*Mindboggle* [42]: Mindboggle’s default algorithm (m = 0) estimates mean, Gaussian and principal curvatures per triangle vertex, based on the relative directions of the normal vectors in a small neighborhood. We choose the optimal radius of neighborhood parameter (n) for each benchmark surface in the same way as for our algorithms (see Section Setting the neighborhood parameter).

## Results

### Quantitative results on benchmark surfaces

#### Calculation of errors

We first evaluate the accuracy of our algorithms using benchmark surfaces with known orientation and curvature. For that purpose, we define two types of errors:

1. For vectors (normals or principal directions):
  $$\begin{array}{c} Vector\;error=1-|{\vec{v}}_{t}\cdot {\vec{v}}_{e}|, \end{array}$$ (19)
  where ${\vec{v}}_{t}$ is a true vector and ${\vec{v}}_{e}$ is an estimated vector for the same triangle, both having length 1. The minimum error is 0, when the true and estimated vectors are parallel, and the maximum error is 1, when the vectors are perpendicular.
2. For scalars (principal curvatures) we use:
  $$\begin{array}{c} Scalar\;relative\;error=|\frac{\kappa_{t}-\kappa_{e}}{\kappa_{t}}|, \end{array}$$ (20)
  where *κ*_*t*_ is a true curvature and *κ*_*e*_ is an estimated curvature for the same triangle. The minimum error is 0, when the estimate equals to the true value, and there is no upper bound to the error.

#### Robust estimation of normals

**Fig 4:**
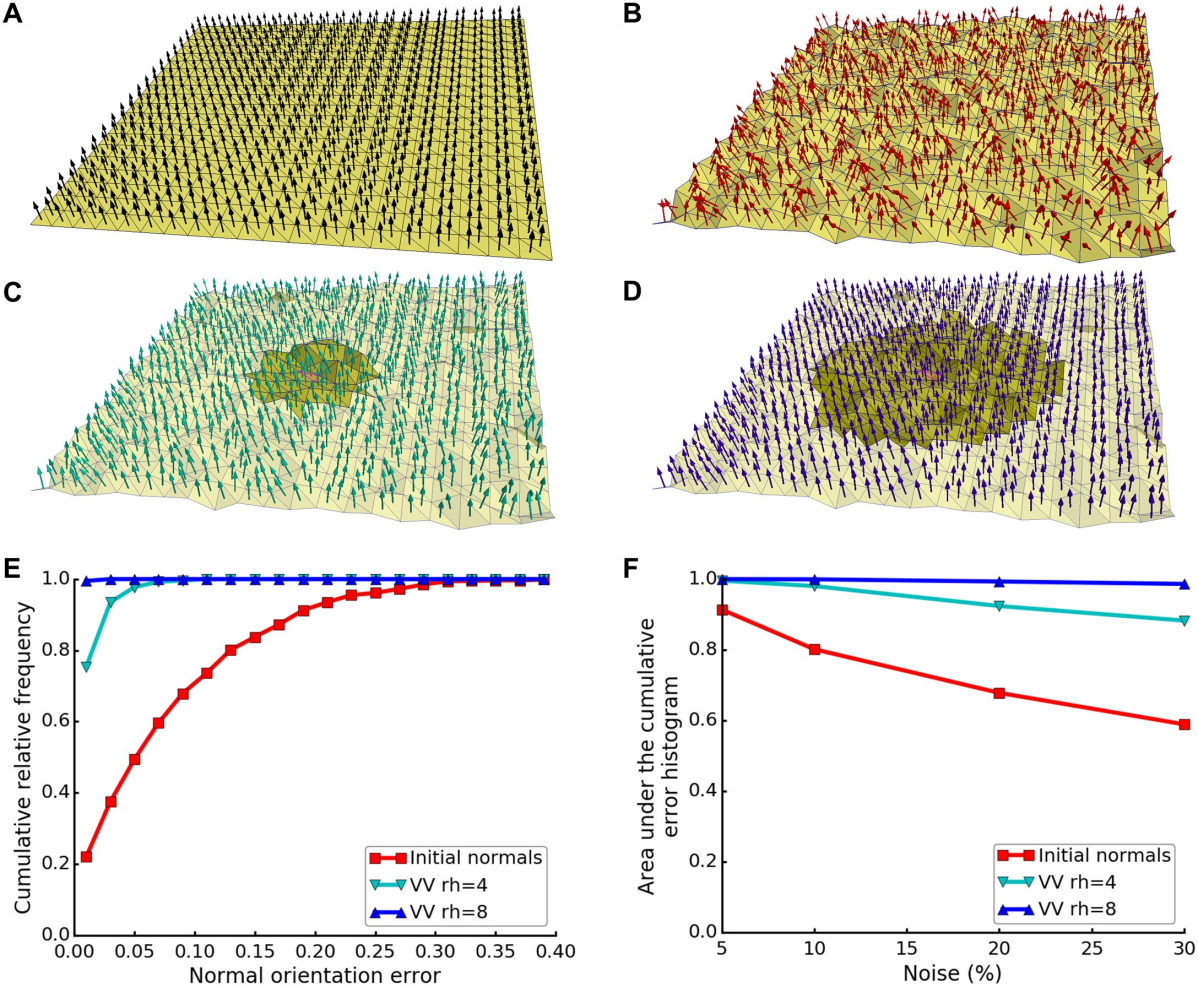
Estimation of normals on a noisy plane. (**A**) True normals (black arrows) on a smooth plane surface (yellow). (**B**) Normals on a noisy plane, where each triangle vertex in the original plane shown in (A) was moved in the direction of its normal vector with Gaussian variance equal to 10% of the average triangle edge. Panels (C-D) show normals on the noisy plane corrected by VV with rh of (**C**) 4 or (**D**) 8 voxels. The neighborhoods of a central triangle are shown in a darker yellow. (**E**) Cumulative relative frequency histogram of normal orientation error for the 10% noisy plane, for initial (uncorrected) normals and those corrected by VV with rh of 4 or 8 voxels. (**F**) Area under the curve of cumulative relative frequency histograms of normal orientation errors, as shown in (E), for planes with different noise levels. Initial normals and those corrected by VV with rh of 4 or 8 voxels are shown. Curve colors in (E-F) correspond to the colors of the normals in (B-D).

Surface normals are required for a reliable estimation of the principal directions and principal curvatures. In this experiment, we wanted to ensure that VV restores the correct orientation of the normals. For this, we used a plane surface with artificially introduced noise to simulate the quantization noise present in surfaces generated from cryo-ET segmentations. The true normals are those from the plane without noise (i.e. parallel to Z-axis, Fig 4A). Noise was introduced to the original plane by moving each triangle vertex in the direction of its normal vector with Gaussian variance equal to e.g. 10% of the average triangle edge. As a result, the triangle normals of the 10% noisy plane were not parallel to each other nor to Z-axis (Fig 4B), which was also reflected by the normal orientation errors up to ∼30% (Fig 4E). Using VV with rh of 4 voxels, the original orientation of the normals was almost restored (Fig 4C), and the errors reduced to below 10% (Fig 4E). Using rh of 8 voxels, the estimation further improved (Fig 4D and 4E), since more neighboring triangles helped to average out the noise. For planes with more noise, the normal orientation errors of the initial normals and the estimated ones with rh of 4 voxels increased, reducing the area under the histogram curve. However, the estimation stayed robust using rh of 8 voxels even for a 30% noisy plane (Fig 4F). Thus, using VV with a high enough rh substantially restores the original orientation of the normals.

#### Setting the neighborhood parameter

As shown above, the size of the neighborhood defined by the rh parameter influences the estimation of normals. Therefore, choosing an appropriate rh for the data is crucial for accurate curvature estimation.

**Fig 5:**
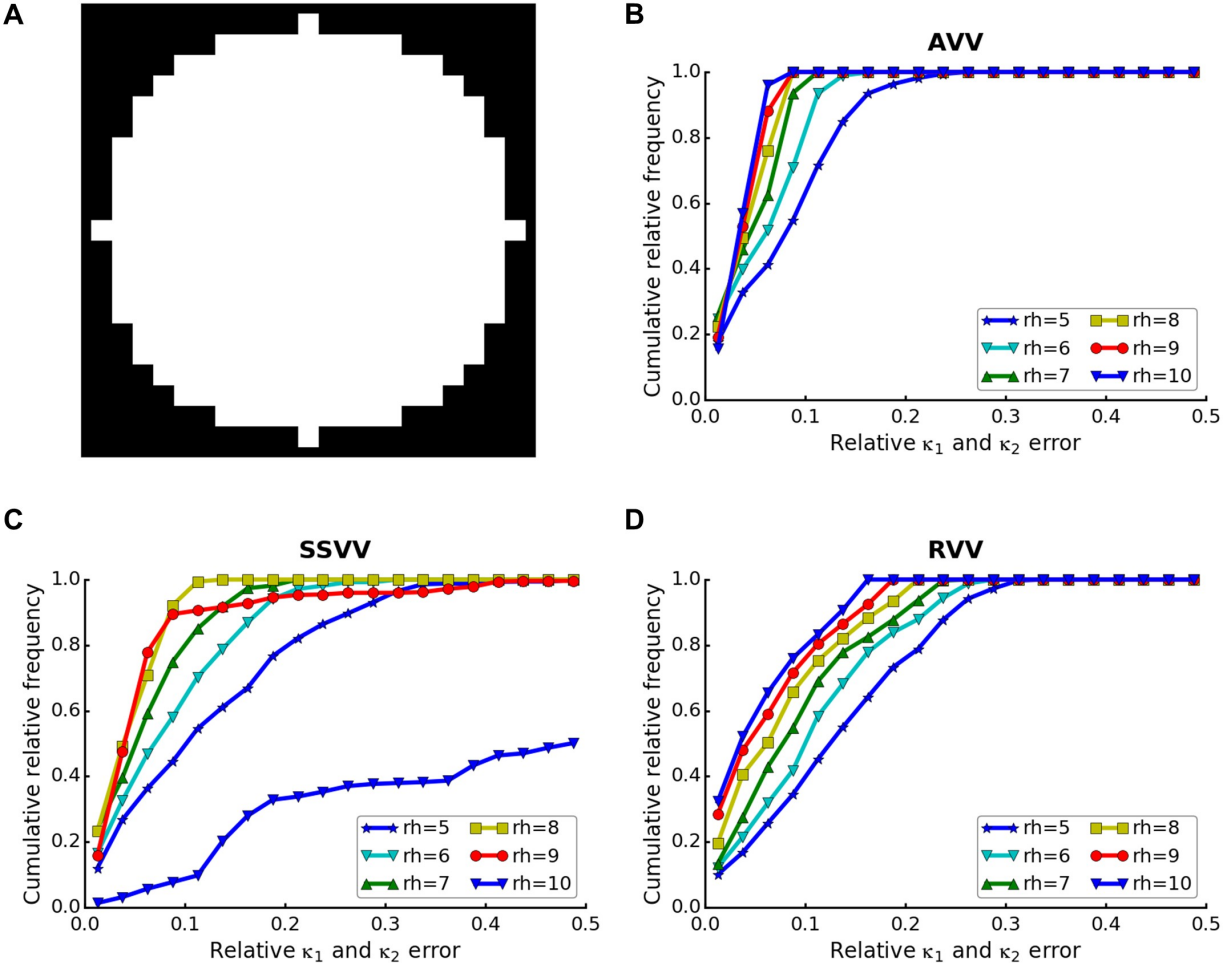
rh parameter choice. (**A**) A central slice of a synthetic segmentation of a sphere with radius = 10 voxels. Panels (B-D) show cumulative frequency histograms of the *κ*_1_ and *κ*_2_ errors on the surface extracted from the segmentation shown in (A), using different rh (5-10 voxels) and algorithms: (**B**) AVV, (**C**) SSVV and (**D**) RVV.

To study the influence of rh in our different curvature estimation algorithms, we generated a synthetic segmentation (25³ voxels) of a sphere with radius of 10 voxels, emulating the quantization noise present in cryo-ET data (the central slice of the sphere is shown in Fig 5A). Then, we generated an isosurface of this segmentation and estimated its curvature using the different algorithms and rh values. We define the optimal rh value for a sphere as the one leading to the least errors in both estimated principal curvatures taken together. As above, we measure the error rate by the normalized area under the cumulative error histograms. For this spherical surface, the lowest errors were reached for rh = 10 voxels for AVV and rh = 8 voxels for SSVV (Fig 5B and 5C, Table 1). These values are close to the sphere radius, suggesting that the most robust estimation can be achieved using a rh approximately similar to the feature radius. Performance of SSVV decreased drastically for rh = 10 voxels, because then less sampling points at exactly this radius lie on the surface, preventing a reliable estimation (Fig 5C, Table 1). Interestingly, the histogram area kept rising until rh = 16 voxels for RVV (Table 1), which exceeds the sphere radius. Actually, the histogram area kept rising even beyond rh = 16 voxels for *κ*_1_ alone, whereas it started to decrease after rh = 12 voxels for *κ*_2_ (Table 1). For practical reasons, we decided to always limit rh by the radius of the feature (Fig 5D).

**Table 1:** rh parameter choice.

rh	RVV			AVV			SSVV		
	*κ*_1_	*κ*_2_	*κ*_1_ and *κ*_2_	*κ*_1_	*κ*_2_	*κ*_1_ and *κ*_2_	*κ*_1_	*κ*_2_	*κ*_1_ and *κ*_2_
5	64.62%	83.59%	74.10%	78.32%	90.68%	84.50%	70.05%	81.97%	76.01%
6	70.21%	87.44%	78.82%	83.80%	94.12%	88.96%	77.85%	89.90%	83.87%
7	74.47%	90.41%	82.44%	87.46%	95.30%	91.38%	83.16%	93.81%	88.48%
8	78.27%	93.24%	85.75%	89.13%	95.67%	92.40%	88.03%	95.46%	**91.75%**
9	80.89%	95.73%	88.31%	90.11%	95.90%	93.00%	87.25%	89.53%	88.39%
10	82.76%	97.24%	90.00%	90.75%	96.09%	**93.42%**	57.63%	2.61%	30.12%
11	84.31%	97.93%	91.12%	90.72%	95.95%	93.34%	0.00%	0.00%	0.00%
12	85.12%	98.23%	91.67%	90.71%	95.46%	93.09%			
13	85.55%	98.09%	91.82%	90.60%	95.14%	92.87%			
14	85.95%	98.01%	91.98%	90.76%	95.08%	92.92%			
15	86.15%	98.01%	92.08%	90.97%	95.02%	92.99%			
16	86.35%	97.92%	**92.13%**	91.14%	94.92%	93.03%			
17	86.53%	97.72%	92.13%	91.28%	94.86%	93.07%			

Performance of our proposed algorithms on noisy sphere with radius = 10 voxels depending on rh (in voxels) is measured by normalized area of the cumulative histograms of the principal curvature errors (separately and taken together). The “*κ*_1_ and *κ*_2_” maxima for each algorithm are shown in bold.

The optimal rh, which can differ between surfaces and algorithms, defines a neighborhood sufficient for robust estimation of curvature. Features with a radius less than rh are averaged (RVV and AVV) or neglected (SSVV), so rh^−1^ can be set as a limit for the maximum curvature that can be reliably computed.

#### Estimation of the curvature sign

**Fig 6:**
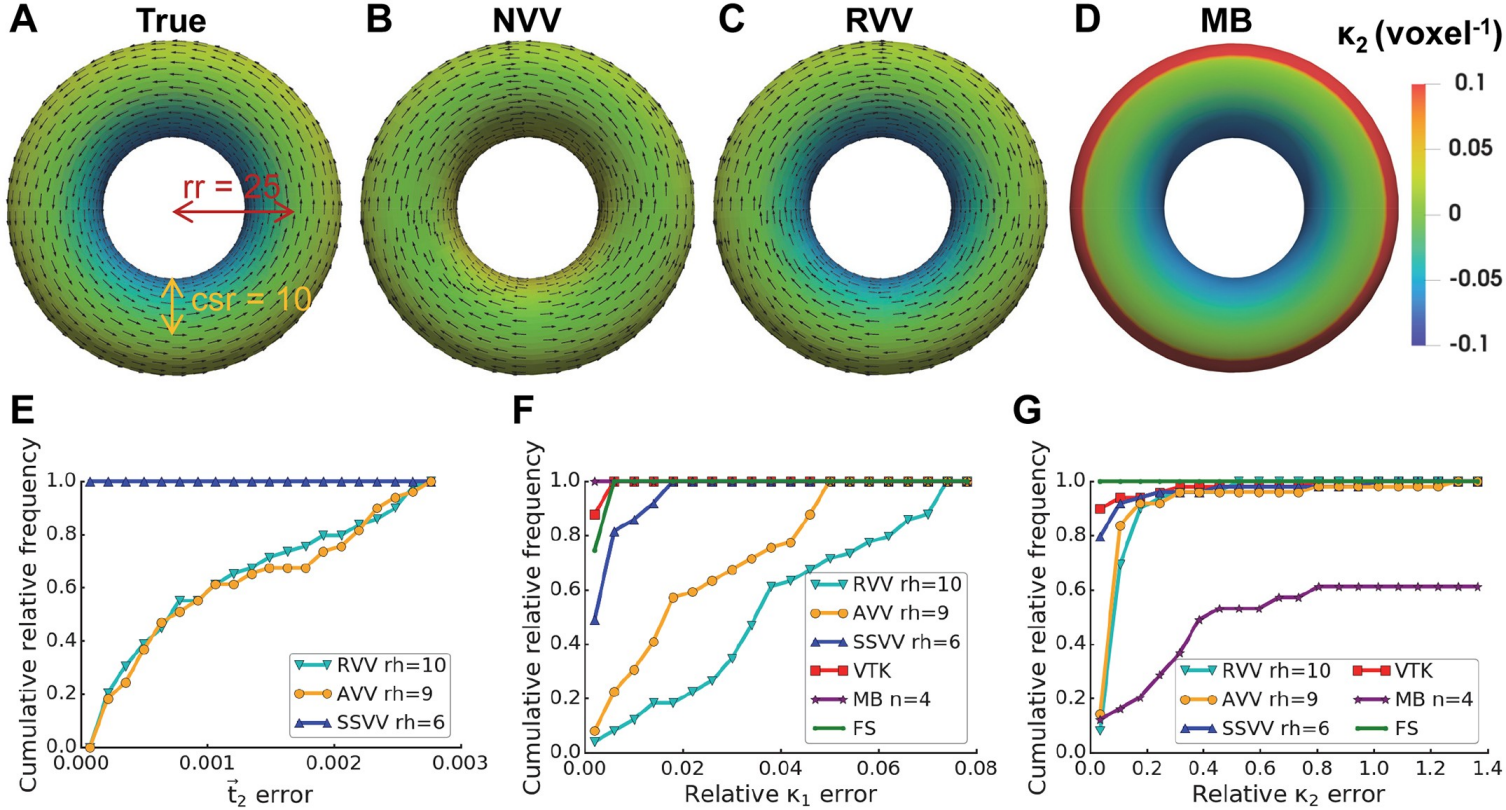
Curvature sign, principal direction and curvature estimation accuracy for a torus. Panels (A-D) show visualizations of *κ*_2_ (voxel^-1^, triangles are color-coded by curvature, see color bar on the right) and ${\vec{t}}_{2}$ (every fourth vector is shown as an arrow from a triangle center): (**A**) true values calculated analytically for a smooth torus surface with ring radius (rr) = 25 voxels and cross-section radius (csr) = 10 voxels, (**B**) estimated values using NVV, (**C**) RVV (both with rh = 8 voxels) and (**D**) Mindboggle (MB, with n = 4 voxels). Panels (E-G) show cumulative relative frequency histograms of the principal direction and curvature errors: (**E**) ${\vec{t}}_{2}$, (**F**) *κ*_1_, (**G**) *κ*_2_ on the torus surface using different algorithms: RVV, AVV, SSVV, VTK, MB and FreeSurfer (FS); the latter three algorithms only for curvatures in (F-G), since they do not estimate principle directions; the optimal rh or n (in voxels) were used for each algorithm and are indicated in the legends.

To determine the correct procedure for curvature sign determination, we used a torus as a benchmark, as this surface has regions with both positive, *κ*_1_*κ*_2_ > 0, and negative, *κ*_1_*κ*_2_ < 0, Gaussian curvature. Analytically calculated *κ*_2_ is shown in Fig 6A. VTK, Mindboggle and FreeSurfer estimated the curvature sign correctly (Fig 6D, 6F and 6G). Whereas NVV did not distinguish negative from positive regions (Fig 6B), RVV and SSVV differentiated these regions correctly (Fig 6C and 6G). Since RVV and SSVV both calculate normal curvature using Eqs 9 and 11, while NVV uses Eq 13, the latter must be the source of the erroneous curvature sign estimation. Therefore, we exclude NVV from further consideration.

#### Accuracy of curvature estimation on smooth surfaces

To evaluate the performance of the different curvature estimation algorithms, we first calculated the errors in principal directions and curvatures using smooth surfaces.

First, we applied the algorithms to the smooth torus surface shown in Fig 6A using for each algorithm an rh optimal for *κ*_2_ (10 voxels for RVV, 9 voxels for AVV and 6 voxels for SSVV). The ${\vec{t}}_{2}$ error histogram is shown in Fig 6E, and very similar results were observed for ${\vec{t}}_{1}$. SSVV estimated both principal directions and curvatures (Fig 6E–6G) more accurately than RVV. AVV slightly outperformed RVV in the estimation of principal curvatures. However, VTK estimated principal curvatures slightly better than the tensor voting-based algorithms for this smooth surface with uniform triangles. Mindboggle with the optimal (for *κ*_2_) n = 4 voxels was the best method for estimating *κ*_1_ (Fig 6F), but the worst for *κ*_2_ (Fig 6D and 6G), whereas FreeSurfer performed the best for *κ*_2_ (Fig 6G). Note that the curvature errors for *κ*_1_ (Fig 6F) were lower than for *κ*_2_ (Fig 6G) for all algorithms. A possible explanation is that *κ*_1_ is constant for a torus and thus easier to estimate, while *κ*_2_ changes depending on the position.

**Fig 7:**
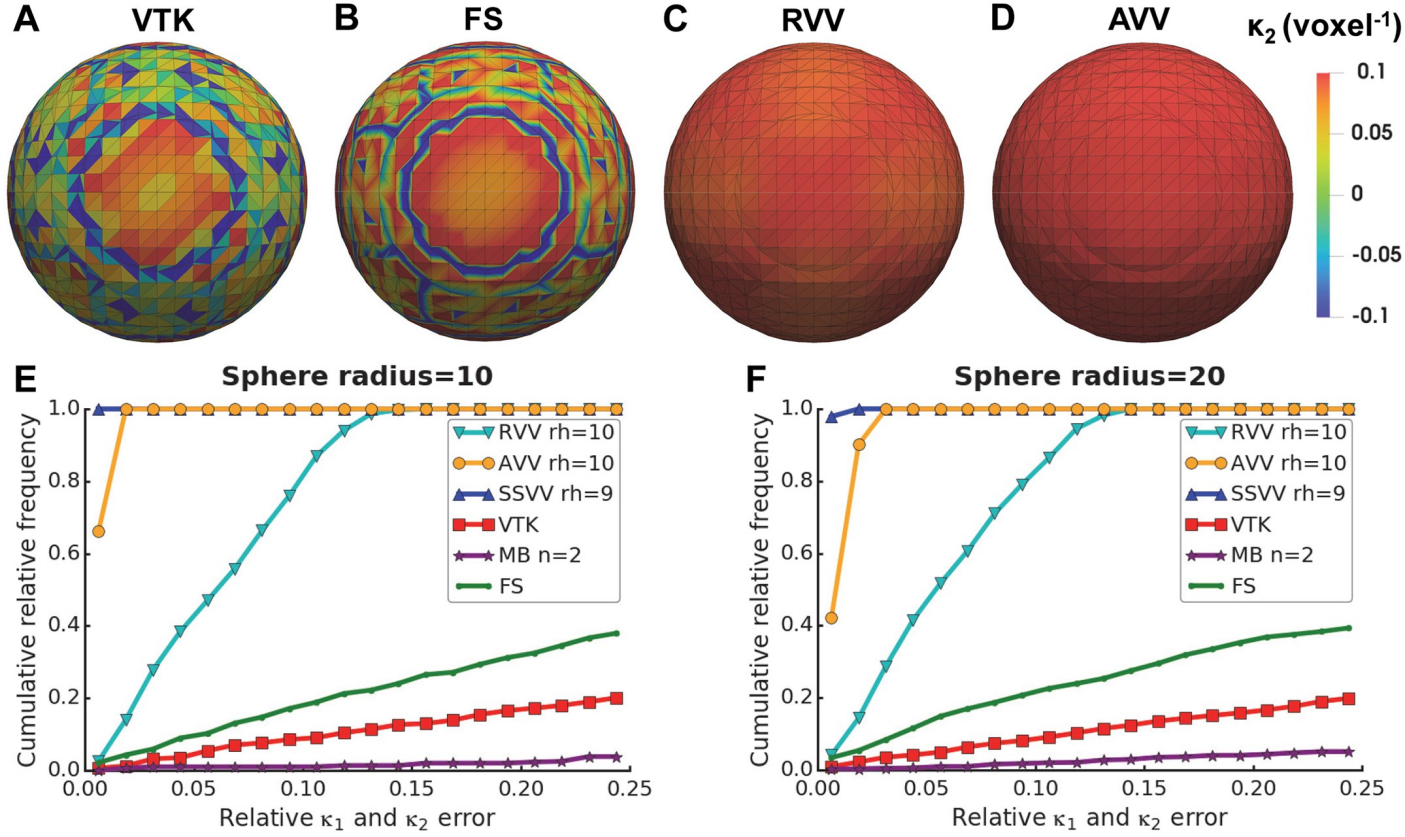
Accuracy of curvature estimation on a smooth spherical surface. Panels (A-D) show visualizations of *κ*_2_ (voxel^-1^) estimated by (**A**) VTK, (**B**) FreeSurfer (FS), (**C**) RVV or (**D**) AVV on a smooth sphere with radius = 10 voxels, using rh = 10 voxels for RVV and AVV. Panels (E-F) show cumulative relative frequency histograms of the principal curvature (*κ*_1_ and *κ*_2_) errors on a smooth sphere with radius = 10 (**E**) or 20 voxels (**F**) using different algorithms: RVV, AVV, SSVV, VTK, Mindboggle (MB) and FS; the values of rh or n (in voxels) used for each algorithm are indicated in the legends.

We also compared the algorithms using a smooth spherical surface with a non-uniform triangle tessellation, generated from a spherical volume mask smoothed using a 3D Gaussian function (*σ* = 3.3) and applying an isosurface. Since both principal curvatures should be the same for a spherical surface, they were considered together. Also, no true principal directions exist for a spherical surface. For a sphere with radius = 10 voxels, the optimal values of rh were used: 10 voxels for RVV and AVV and 9 voxels for SSVV as well as the optimal n = 2 voxels for Mindboggle. VTK, Mindboggle and FreeSurfer had very high errors (Fig 7A, 7B and 7E). The maximum error was only ∼0.16 for RVV (Fig 7C and 7E), while AVV achieved a substantial improvement (maximum error ∼0.03) over RVV (Fig 7D and 7E), presumably because of the non-uniform tessellation of the sphere. SSVV performed slightly better than AVV (maximum error ∼0.01; Fig 7E).

To test how stable the algorithms are for different curvature scales, we increased the radius of the smooth sphere from 10 to 20 voxels, while leaving the rh and n values the same. All algorithms performed almost the same as for the sphere with radius 10 voxels (Fig 7F).

Altogether, the evaluation results on smooth benchmark surfaces show that the tensor voting-based algorithms are quite stable to feature sizes variations (beyond rh) and irregular triangles within one surface (Fig 7), whereas VTK, Mindboggle and FreeSurfer only perform well for a very smooth surface with a regular triangulation (Fig 6). AVV can deal with non-uniformly tessellated surfaces better than RVV, likely because curvature votes are also weighted by relative triangle area in AVV. In the original algorithm [33], weighting curvature votes by triangle area would not make sense because normals and curvatures are estimated at triangle vertices. Since we decided to estimate normals and curvatures at triangle centers instead, curvature votes are cast by complete triangles and weighting them by triangle area proved to be advantageous.

#### Robustness to surface noise

Surfaces generated from segmentations of biological membranes are not smooth, as the surface triangles tend to follow the voxel boundaries resulting in steps. As we are considering binary voxel values, the size of the steps depends on the voxel size of the segmented tomogram.

**Fig 8:**
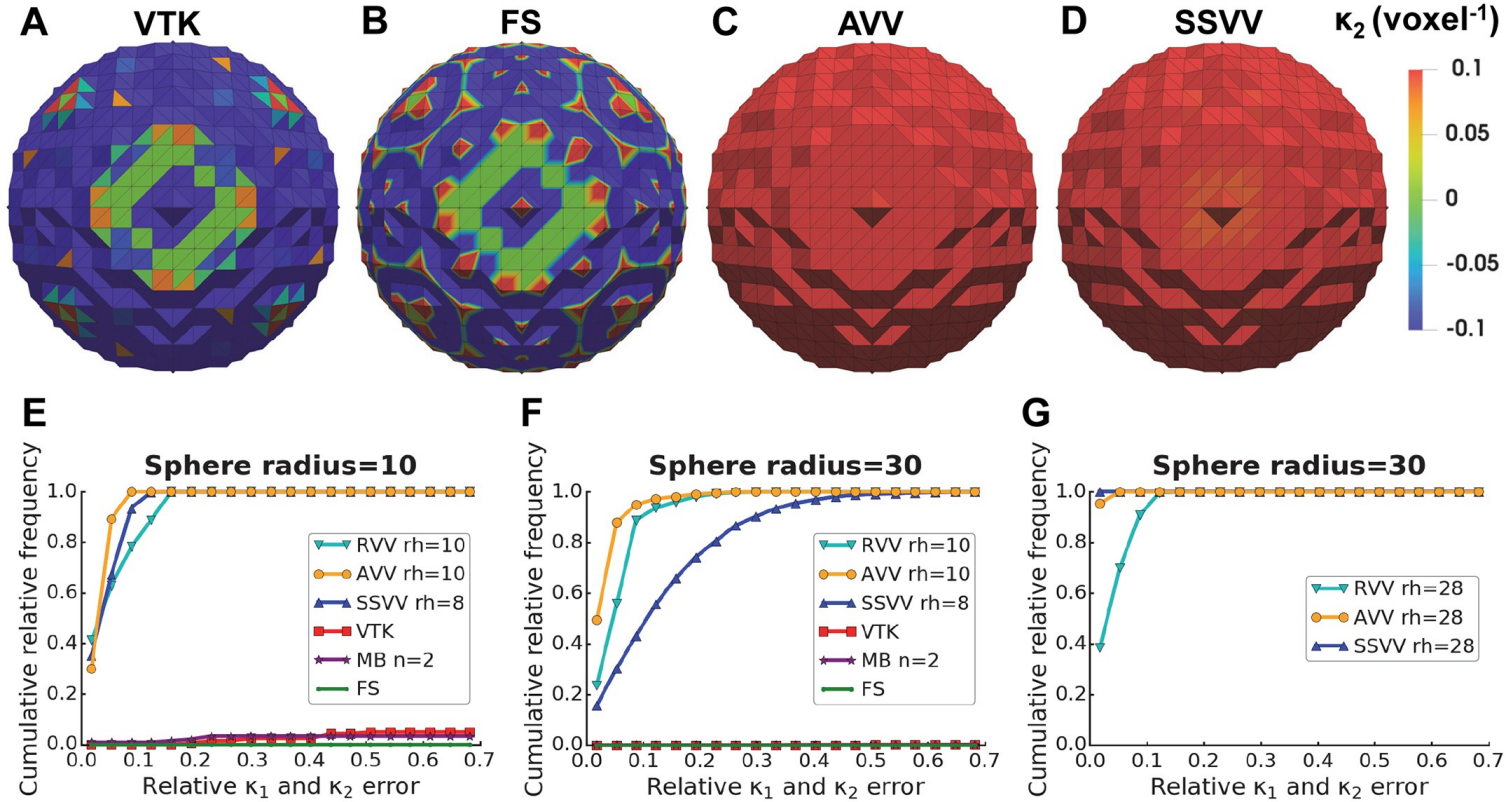
Accuracy of curvature estimation on a spherical surface with quantization noise. Panels (A-D) show visualizations of *κ*_2_ (voxel^-1^) estimated by (**A**) VTK, (**B**) FreeSurfer (FS), (**C**) AVV using the optimal rh = 10 voxels or (**D**) SSVV using the optimal rh = 8 voxels on a sphere with quantization noise and radius = 10 voxels. Panels (**E**-**G**) show cumulative relative frequency histograms of the principal curvature (*κ*_1_ and *κ*_2_) errors on a spherical surface with quantization noise and (E) radius = 10 or (F-G) 30 voxels using RVV, AVV, SSVV, VTK, Mindboggle (MB) and FS; latter three algorithms only in (E-F). The values of rh or n (in voxels) used for each algorithm are indicated in the legends.

To test how the algorithms perform in presence of quantization noise, we generated a step-like surface of a sphere with a radius of 10 voxels, as in Fig 5. As expected, VTK only measured the curvature differences between directly neighboring triangles, resulting in high errors, similar to Mindboggle (using the optimal n = 2 voxels) and FreeSurfer (Fig 8A, 8B and 8E). To compare RVV, AVV and SSVV, we first used optimal rh values (10 voxels for RVV and AVV and 8 voxels for SSVV, Fig 5B–5D, Table 1). The principal curvature errors were higher for AVV and SSVV compared to the smooth sphere (5-10 fold, compare X-axis scales in Figs 7E and 8E), but were similar for RVV. However, AVV outperformed SSVV in this case (Fig 8C–8E), whereas the latter performed slightly better on the smooth spherical surface (Fig 7E).

We compared again the accuracy of the algorithms for increasing feature size and a constant rh value. When using a sphere with a radius of 30 voxels, VTK, Mindboggle and FreeSurfer still performed extremely poorly, and the estimation accuracy of SSVV decreased drastically, while the performance of AVV and RVV decreased only slightly (Fig 8F). The performance of SSVV improved using a rh value similar to the sphere radius, 28 voxels, which should be close to optimum (Fig 8G). Also the performance of AVV increased in this case, while the performance of RVV stayed similar (compare Fig 8F and 8G).

Therefore, when quantization noise is present, all our algorithms perform better than the currently available methods tested here. SSVV requires a higher rh in the range of the curvature radius, while AVV is quite stable with a lower rh value. Using a very high rh is generally not advisable, as it would lead to the underestimation of curvatures at smaller surface features. Since RVV performed consistently worse than AVV, we exclude RVV from further comparison.

#### Higher errors at surface borders

As explained previously, membranes in cryo-ET segmentations have holes and open ends. Thus, we aimed for a curvature estimation algorithm that is robust to such artifacts.

Tensor voting-based algorithms use a supporting neighborhood in order to improve the estimation, so holes much smaller than the neighborhood region do not affect them critically. However, a vertex close to surface border has considerably less neighbors. Therefore, we hypothesized that the estimation accuracy at vertices close to such borders would be worse. To prove this hypothesis, we generated a smooth cylindrical surface opened at both sides with radius = 10 and height = 25 voxels and evaluated the performance of our algorithms. Optimal rh values were used for AVV (5 voxels) and for SSVV (6 voxels), as well as optimal n for Mindboggle (2 voxels). FreeSurfer was not included in this test, since it failed on this open surface.

**Fig 9:**
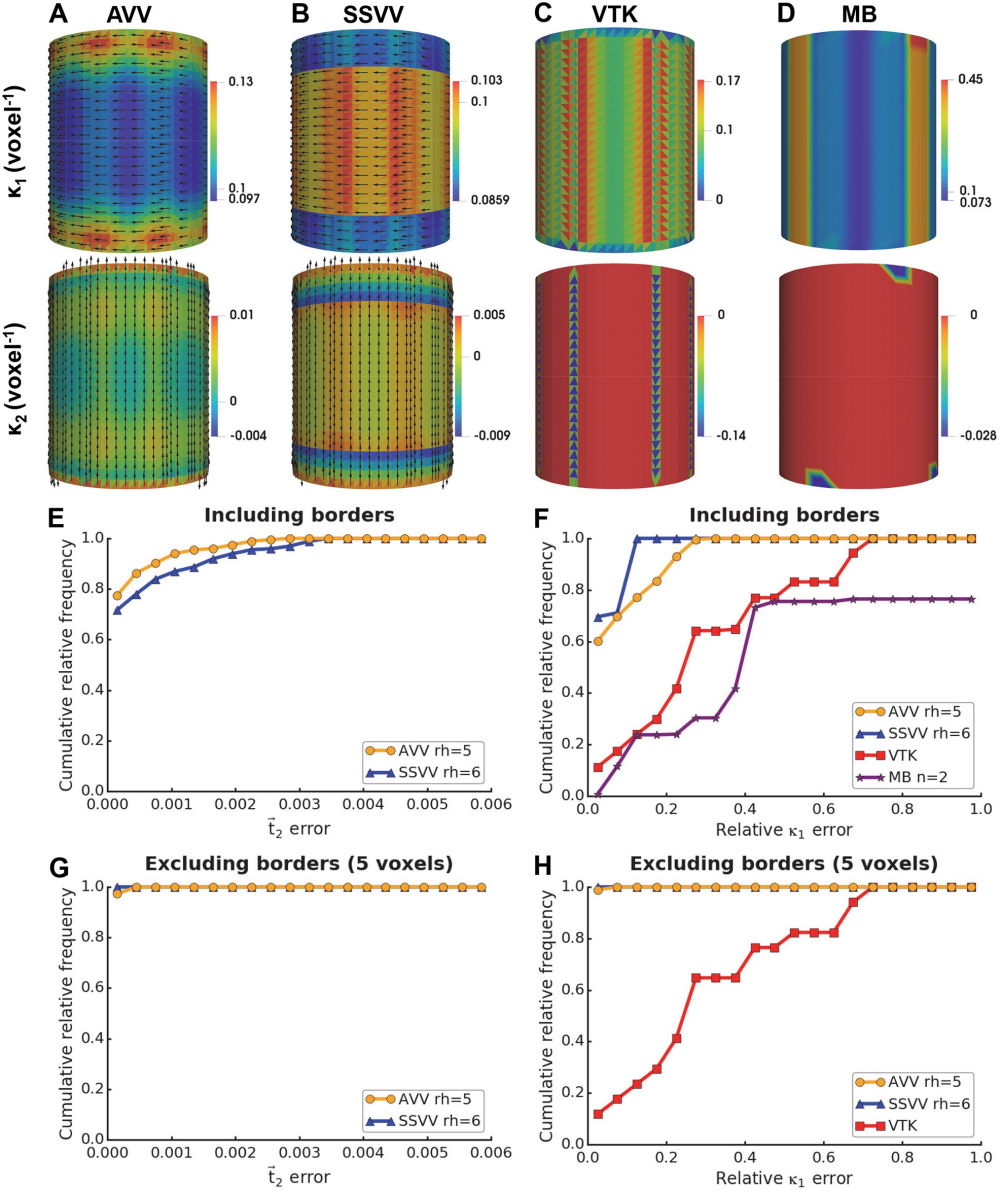
Estimation accuracy on a cylindrical surface. Panels (A-D) show principal curvatures on a smooth cylinder with radius = 10 and height = 25 voxels estimated by different algorithms: (**A**) AVV using the optimal rh = 5 voxels, (**B**) SSVV using the optimal rh = 6 voxels, (**C**) VTK and (**D**) Mindboggle (MB) using the optimal n = 2 voxels; the estimated *κ*_1_ and *κ*_2_ are shown in their original ranges; true *κ*_1_ = 0.1, true *κ*_2_ = 0 voxel^-1^. Panels (E-H) show cumulative relative frequency histograms of the ${\vec{t}}_{2}$ errors (**E**, **G**) and *κ*_1_ errors (**F**, **H**) on the cylinder using different algorithms: AVV, SSVV, VTK and MB; latter two algorithms only for *κ*_1_ in (F, H); the optimal rh or n (in voxels) were used for each algorithm and are indicated in the legends. In panels (G-H), values within 5 voxels to the graph border were excluded.

As expected, both tensor voting-based algorithms made a worse estimation near borders: AVV overestimated *κ*_1_ gradually when moving towards the borders and *κ*_2_ at the last triangles (Fig 9A), while SSVV underestimated *κ*_1_ consistently and made a gradient of wrong estimations for *κ*_2_ in the same region (Fig 9B). Since VTK does not use a bigger neighborhood, it showed high errors at changes in the triangle pattern all over the cylinder (Fig 9C). Mindboggle showed high errors for *κ*_1_ in a striped pattern and for *κ*_2_ at some patches near the borders (Fig 9D). SSVV and AVV showed ${\vec{t}}_{2}$ and *κ*_1_ errors in the same range (Fig 9E and 9F), while VTK and Mindboggle made higher *κ*_1_ errors than our algorithms (Fig 9F). When excluding values within the distance of 5 voxels to the graph border, the errors were virtually eliminated for AVV and SSVV, but did not change for VTK (Fig 9G and 9H). For Mindboggle, we could not exclude values at borders because our graph structure used for borders filtering is not available for that method. However, as one can see in Fig 9D, the high *κ*_1_ errors of Mindboggle would not have been eliminated with this strategy.

Altogether, these benchmark results demonstrate the validity of our tensor voting-based algorithms and their robustness to quantization noise, especially of AVV. Additionally, curvature estimations at surface borders can be erroneous, so they should be excluded from an analysis.

### Application to biological surfaces

#### Choice of algorithms and parameters for membranes from cryo-ET

AVV and SSVV proved most robust to quantization noise in synthetic surfaces. To evaluate their performance on real cryo-ET data, we used a cER compartment segmentation that contains several high curvature regions or peaks [16].

**Fig 10:**
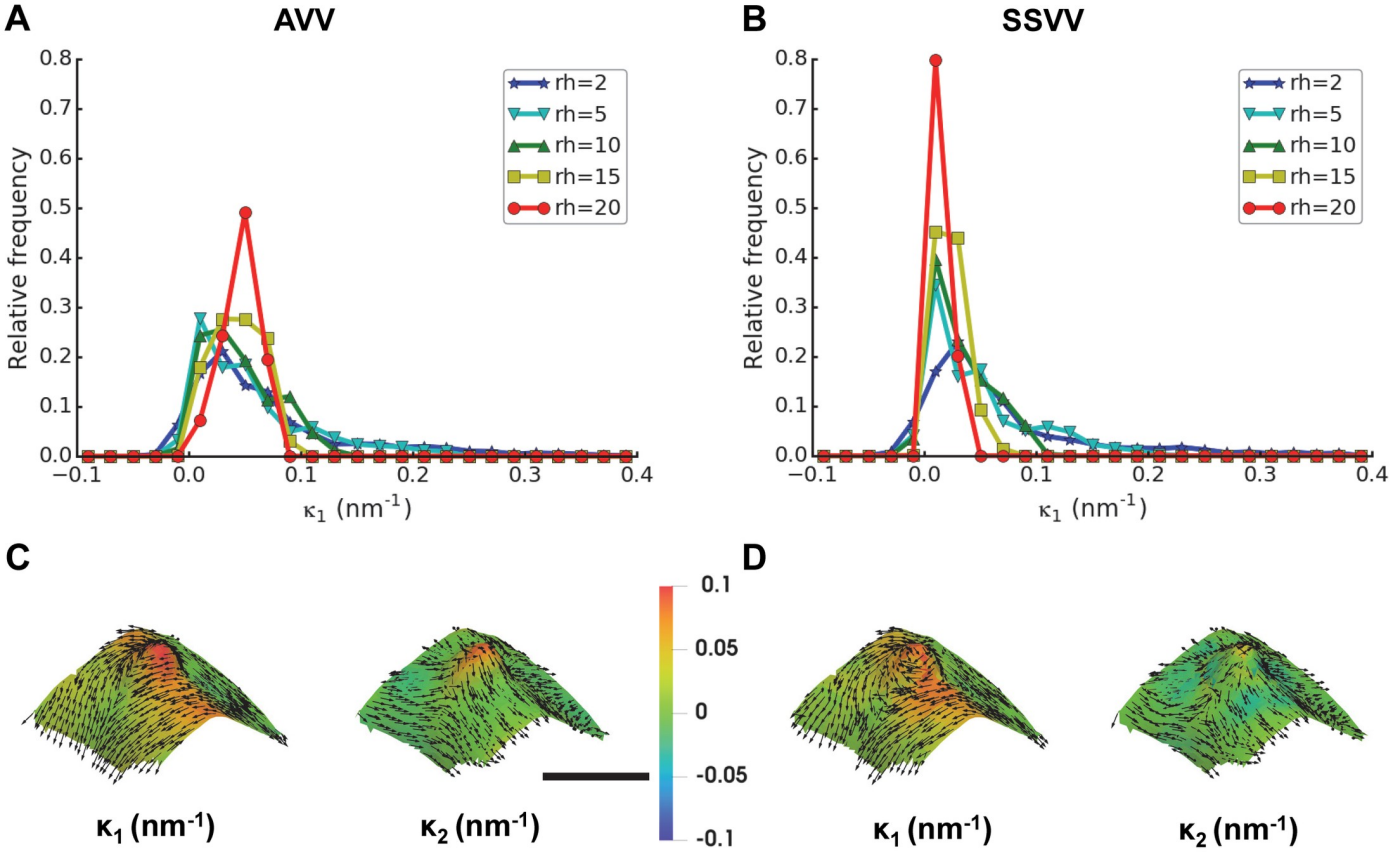
Algorithms and parameters comparison using a small cER membrane feature from cryo-ET. Surface of a cER membrane region with maximum base radius ∼10 nm (from the same tomogram of a yeast cell as the cER in Fig 11) was generated using the compartment segmentation. Panels (A-B) show relative frequency histograms of *κ*_1_ estimated by (**A**) AVV or (**B**) SSVV using rh = 2, 5, 10, 15 and 20 nm. Panels (C-D) show visualizations of the estimated *κ*_1_ and *κ*_2_ (color scale was set to the value range of [-0.1, 0.1] nm^-1^ in both panels) and the corresponding principal directions (black arrows, sampled for every forth triangle) by (**C**) AVV or (**D**) SSVV using rh = 10 nm (scale bar: 20 nm, applies for both panels).

First, we studied the relationship between the rh parameter and the feature size. For this, we isolated a single cER peak with a maximum base radius of approximately 10 nm from a tomogram and estimated its curvature using several rh values. We observed that real membrane features have a diverse curvature distribution with several local maxima and minima (Fig 10A and 10B). For low values of rh, the distributions of curvature are broad, getting progressively sharper with increasing rh. For AVV (Fig 10A), the maximum amount of values around 0.1 nm^-1^ (corresponding to the 10 nm radius of the peak) is reached for rh = 10, indicating that the feature is observed as a whole and its smaller components fade. Higher rh no longer produce curvature values around 0.1 nm^-1^, indicating that the feature is averaged out. A similar trend is observed for SSVV (Fig 10B), although this method produces less curvature values around 0.1 nm^-1^, thus underestimating the real curvature of the feature.

Second, we visualized the principal curvatures of the feature using rh = 10 nm to analyze the difference between the two curvature estimation algorithms. For this specific feature, its principal curvatures estimated by AVV (Fig 10C) increased in the direction from the base to the summit, while SSVV (Fig 10D) underestimated the curvatures, especially *κ*_2_. Since SSVV sampled only surface points at distance equal to rh of 10 nm from each triangle center, it “oversaw” the high curvature at and near the summit. Contrary to SSVV, AVV considered all triangles within the geodesic neighborhood and thus estimated the curvature increase towards the summit correctly. This example confirms that AVV performs better than SSVV for complex surfaces like biological membranes.

**Fig 11:**
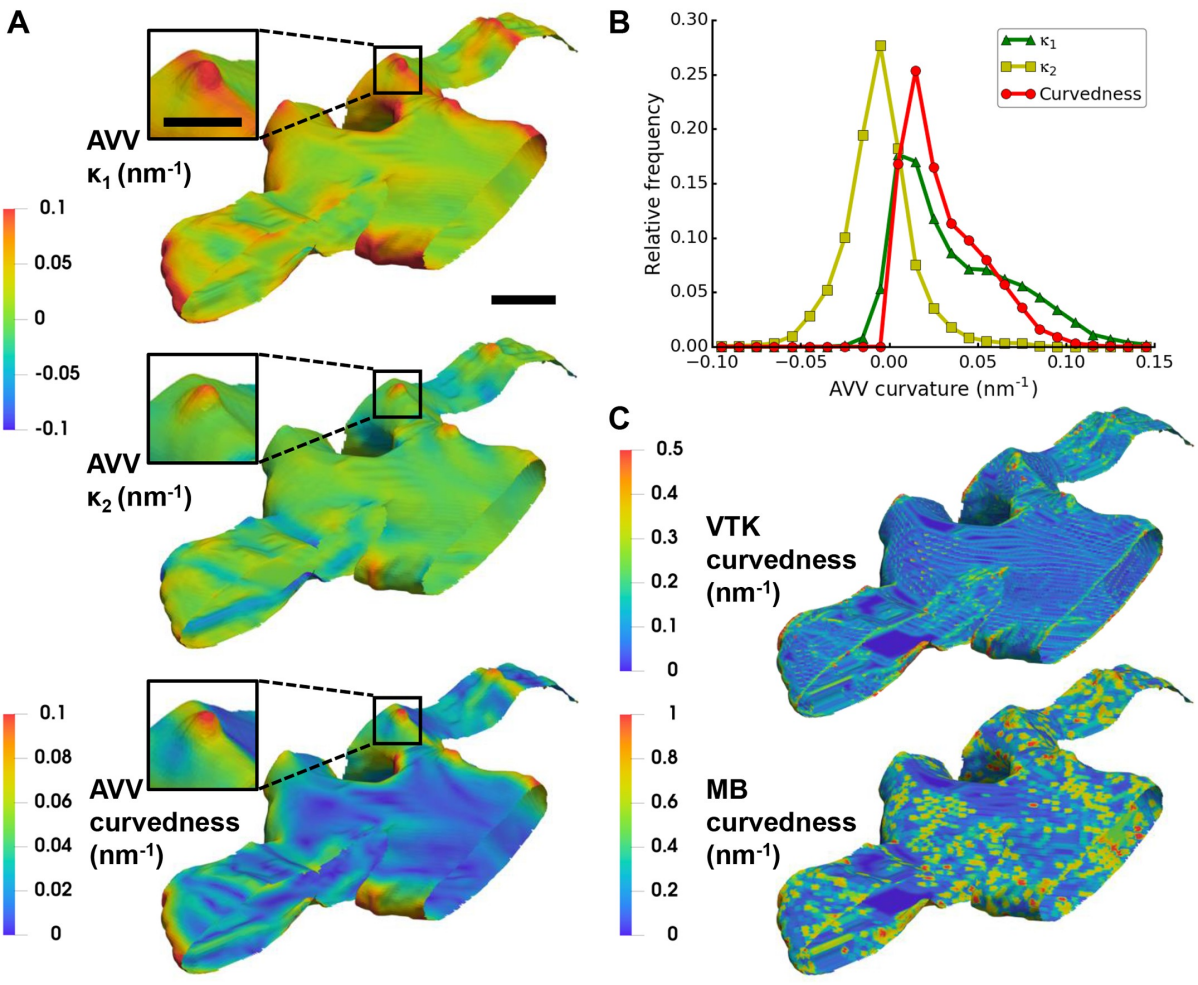
Application of curvature estimation algorithms to a cER membrane from cryo-ET. Analysis of yeast cER membrane curvature on a surface generated using the compartment segmentation. (**A**) Visualizations of the curvatures: *κ*_1_, *κ*_2_ and curvedness, estimated by AVV with rh of 10 nm (scale bar: 40 nm). The insets show the peak feature from Fig 10 (scale bar: 20 nm). Color scale was set to the value range of [-0.1, 0.1] nm^-1^ for *κ*_1_ and *κ*_2_ and of [0, 0.1] nm^-1^ for curvedness. (**B**) Relative frequency histograms of the curvatures estimated by AVV shown in (A). (**C**) Visualizations of curvedness on the same surface as in (A) calculated by VTK (top) and Mindboggle (MB; using n = 2 nm; bottom), scale bar as in the main panel (A). Since curvedness ranges were larger for these algorithms, color scales were set to the value range of [0, 0.5] nm^-1^ for VTK and [0, 1] nm^-1^ for Mindboggle. The tomogram and segmentation are deposited in EM Data Bank (EMD-10765).

Lastly, we applied AVV with rh = 10 nm to the full cER membrane surface, from which the peak shown in Fig 10 was extracted (Fig 11A and 11B). For comparison, we also applied VTK and Mindboggle to this surface (Fig 11C). Visually, n = 2 nm yielded the best results for Mindboggle. On this membrane surface, AVV clearly outperforms VTK and Mindboggle, which provide very noisy results with high values following the steps between neighboring triangles and the surface borders (compare the values of curvedness in Fig 11A and 11C).

#### Curvature comparison across cellular organelles

**Fig 12:**
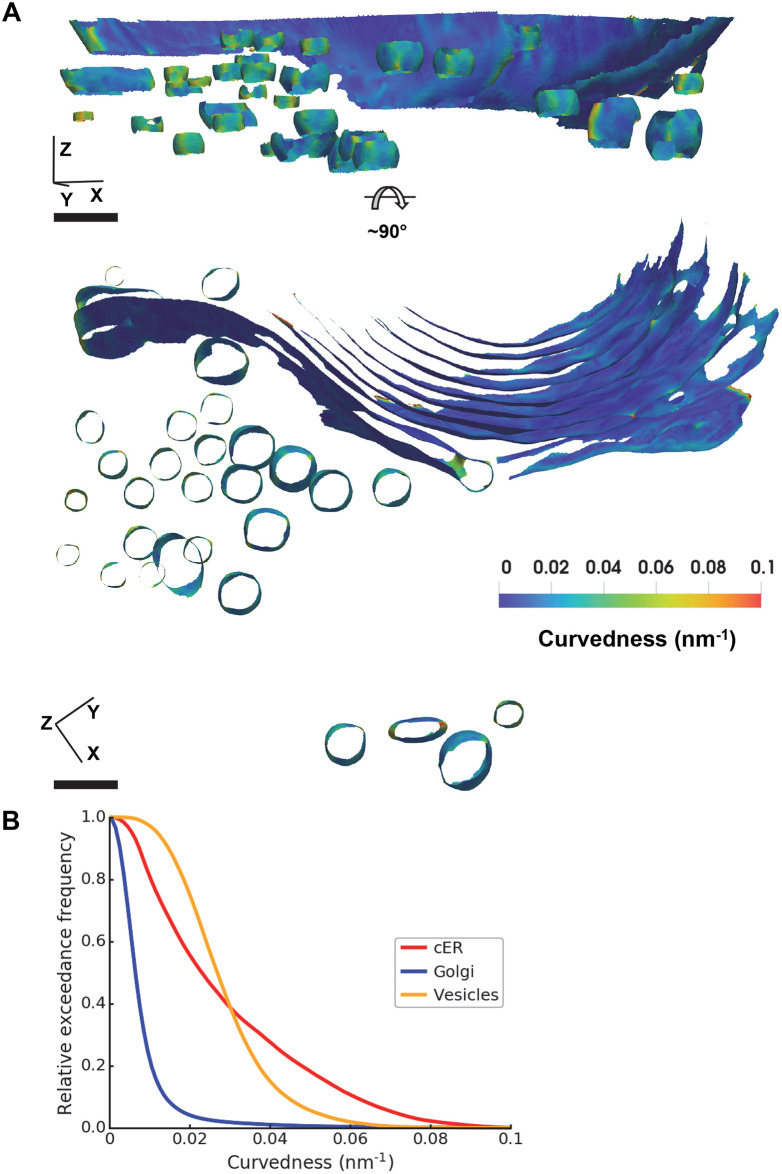
Application of AVV to Golgi and vesicles from cryo-ET. (**A**) Two different views of surfaces of Golgi and vesicles (from a primary mouse neuron) generated using the compartment segmentation showing curvedness estimated by AVV with rh of 10 nm. Color scale was set to the value range of [0, 0.1] nm^-1^ (scale bar: 100 nm). (**B**) Relative exceedance frequency histograms (reversed cululative histograms with frequency normalized to the total surface area of each compartment) of the curvedness of cER (shown in Fig 11A and 11B), Golgi and vesicles (shown in panel A of this figure), excluding values within 1 nm to surface border. The tomogram and segmentations are deposited in EM Data Bank (EMD-10766).

To test our method on membranes with different morphologies, we segmented the Golgi apparatus and Golgi-derived vesicles in a tomogram recorded on a mouse neuron. A Golgi apparatus is composed of flat cisternae stacked in a bent, semicircular shape. Again, we extracted the membrane surfaces using the compartment segmentation and estimated the curvatures using AVV with rh of 10 nm (Fig 12A). To minimize borders effects, values within 1 nm to surface border were excluded for plotting. Fig 12B compares the curvedness of the cER (Fig 11) with that of the Golgi and Golgi-derived vesicles. The histogram shows that the Golgi has much lower curvedness than the other two organelles, whereas the cER reaches higher curvedness values than the vesicles. The results can be visually confirmed: the thin and long Golgi cisternae are only slightly curved, while the vesicles are smaller and thus much more curved (Fig 12A). The cER is generally less curved than the vesicles, but has high curvature at the peaks and sides of its sheets (Fig 11A). These data show that curvedness estimated by AVV can be a useful descriptor of biological membranes.

#### Application to other data types

**Fig 13:**
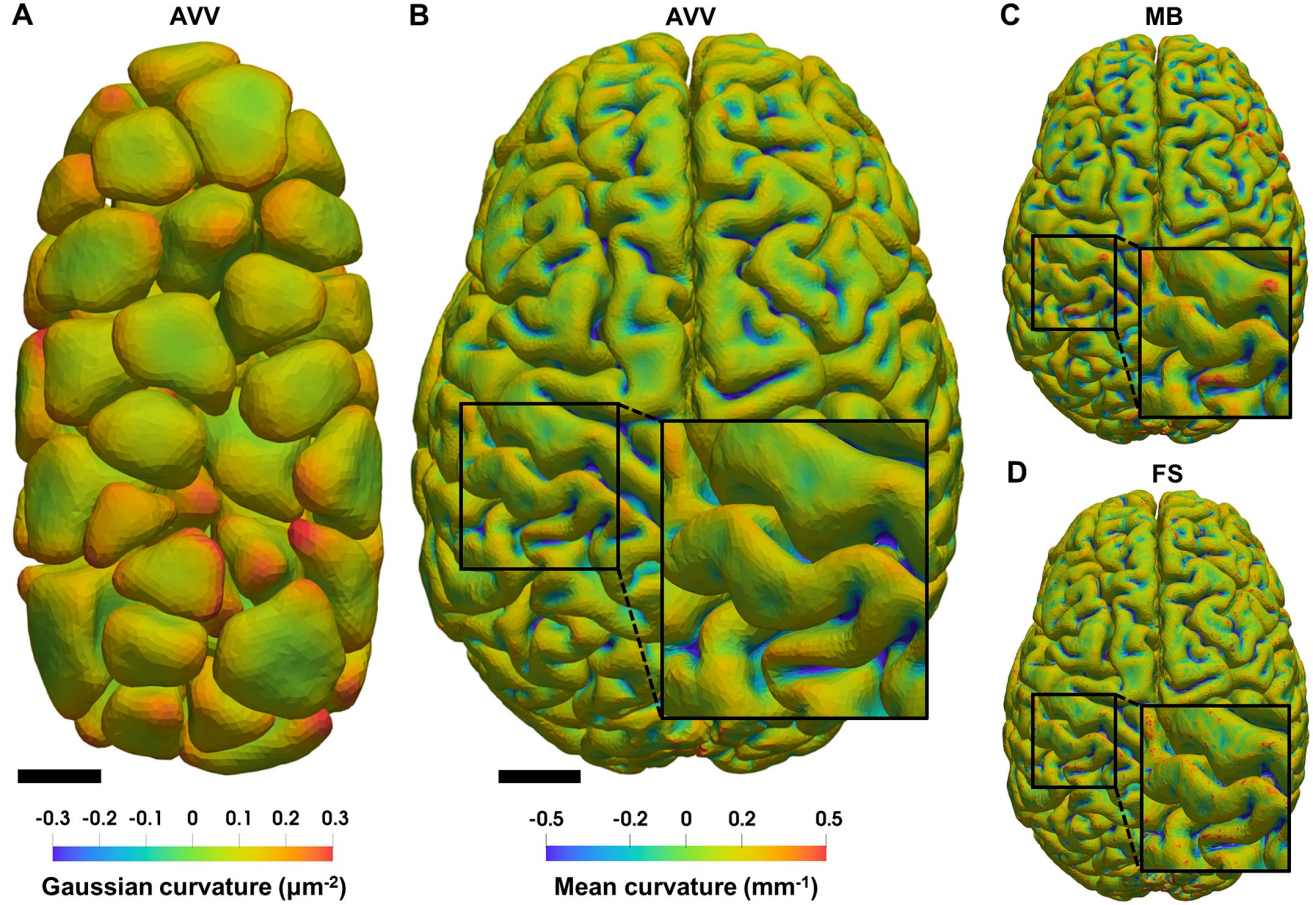
Application of curvature estimation algorithms to other data types. (**A**) Surfaces of *C. elegans* embryo cells imaged by confocal light microscopy and segmented by LimeSeg [22], colored by Gaussian curvature (*μ*m^-2^) estimated by AVV using rh = 3 *μ*m (scale bar: 5 *μ*m). (**B**) Cortical pial surfaces of both human brain hemispheres imaged by MRI and segmented by FreeSurfer [52], colored by mean curvature (mm^-1^) estimated by AVV using rh = 2 mm (scale bar: 20 mm). Panels (C-D) show the same brain surface colored by mean curvature (mm^-1^) with the same color scale as in (B) estimated by (**C**) Mindboggle (MB; using n = 2 mm) and (**D**) FreeSurfer (FS).

To demonstrate the applicability of AVV beyond cryo-ET, we applied it to two other data types. The first data set is comprised of *C. elegans* embryo cells imaged by confocal light microscopy and segmented by LimeSeg [22]. The cell surfaces colored by their Gaussian curvature estimated by AVV using rh = 3 *μ*m are shown in Fig 13A. The second data set, taken from Mindboggle [42], are cortical pial surfaces of both human brain hemispheres imaged by MRI and segmented by FreeSurfer [52]. The cortical surfaces colored by their mean curvature estimated by AVV using rh = 2 mm are shown in Fig 13B. The range of curvature values for the embryo and the brain is consistent with their sizes. Using Mindboggle [42] with n = 2 mm (Fig 13C) and FreeSurfer [41] (Fig 13D), we obtained comparable, but noisier, mean curvature distributions on the brain; FreeSurfer introduced finer-grained noise than Mindboggle. Despite the lack of ground truth, this comparison suggests that AVV provides a more accurate curvature estimation for different data types.

### Implementation and availability

All the described algorithms and the tests on benchmark surfaces were implemented using Python and are available in PyCurv at https://github.com/kalemaria/pycurv, along with the experimental data sets and scripts allowing to obtain the results presented in the Section Application to biological surfaces. PyCurv depends only on open source packages, including: Pyto [53], Graph-tool [48] and VTK [30]. Note that FreeSurfer [52] and Mindboggle [42] had to be installed and called externally for the evaluation; FreeSurfer version “stable v6.0.0” for Linux and Mindboggle Docker container from 2019-09-24 were used.

## Discussion

In this article, we described a method for the estimation of the local curvature of biological membranes and validated it on synthetic and real data. The curvature estimation workflow in PyCurv can be divided in two main steps. The first step is to represent the membrane as a triangle mesh surface that can be obtained from two different types of segmentation: segmentation of the membrane alone, or a filled segmentation of a membrane-bound cellular compartment. The second option usually demands more human intervention but the surface orientation could be recovered perfectly in our experiments. Smoothing of the filled segmentation prior to surface extraction leads to less quantization noise because the surface is extracted at subvoxel precision. Surface triangles are mapped to a graph to facilitate the computation of geodesic distances and to filter border artifacts. The second step is to determine the underlying surface orientation (represented by normal vectors), local curvatures and principal directions.

Here, we evaluated the performance of our curvature estimation algorithms, RVV, AVV and SSVV (adaptations of [33] and [40]) against the publicly available VTK [30], FreeSurfer [41] and Mindboggle [42]. Although we chose the optimal radius of the neighborhood (n) parameter for each benchmark surface, Mindboggle performed poorly on irregular and noisy surfaces. Also FreeSurfer, which performed the best on a smooth and regular surface, yielded high errors on irregular and noisy surfaces. Moreover, FreeSurfer cannot be applied to surfaces containing borders, so it is not applicable for cryo-ET data. Our tests using synthetic and biological surfaces showed that the proposed algorithms, RVV, AVV and SSVV, are more robust to quantization noise than the above-mentioned existing methods. AVV performs better than RVV for non-uniformly tessellated surfaces. For complex non-spherical surfaces like biological membranes, AVV yields better results than SSVV. Therefore, AVV is the default algorithm in PyCurv.

Curvature is a local property, so its value on discrete surfaces depends on the definition of a neighborhood. Robustness to noise increases with the neighborhood size by averaging the contributions of the neighboring triangles. However, features smaller than the neighborhood are averaged out. Therefore, the neighborhood size defines the scale of the features that can be analyzed. To achieve more reliable results for cryo-ET segmentations that contain holes, curvature values at surface borders and/or higher than rh^-1^ should be excluded from the analysis.

PyCurv was already applied in a cryo-ET study in yeast proposing that cER membrane curvature plays a key role in the regulation of ER-to-PM lipid homeostasis at membrane contact sites [16]. Moreover, the analysis of data generated by MRI and light microscopy shows that our method can be applied to any segmented membrane compartments or other volumes from which a surface can be extracted, originating from any 3D imaging technique. We conclude that the open-source Python package PyCurv can be used to reliably process cryo-ET and other data to study membrane and surface curvature in a large variety of applications.

## Supporting information

S1 Video PyCurv workflow. The visualization of PyCurv processing workflow, as described in Fig 1A, for the tomogram from Fig 2, showing in the order of occurrence: *membrane* and *compartment segmentations* of the cortical ER, generated surface, normals estimated by Vector Voting (VV) and curvedness estimated by Augmented Vector Voting (AVV) algorithms.
(MP4)
